# Design and implementation of an inductor based cell balancing circuit with reduced switches for Lithium-ion batteries

**DOI:** 10.1038/s41598-024-80096-9

**Published:** 2024-11-20

**Authors:** R. Venkatasatish, C. Dhanamjayulu

**Affiliations:** School of Electrical Engineering, Vellore Institute of Technology, Vellore, Tamil Nadu India

**Keywords:** Energy science and technology, Engineering

## Abstract

Electrical Vehicles (EVs) will eventually lead to reduced availability of fossil fuels and increased asset demand. The efficiency and range of electrically powered vehicles are influenced by the battery. The chemical structure of lithium-ion (LIB) batteries is particularly vulnerable to overcharging and deep discharge, which may damage the battery, reduce its life, and even cause dangerous things. Hence an efficient management system known as a battery management system (BMS) is needed to balance, protect, and manage the energy of the battery pack. Cell balancing is the most important of the three in terms of the longevity of the battery structure. Cells in a battery pack are imbalanced during charging and discharging due to the design parameters of cells in a battery pack which results in battery degradation and an increase in temperature. Hence efficient cell balancing techniques are needed to balance the battery pack to improve the safety level and life. Hence, the paper proposed a novel 2-layer multi-inductor active cell balancing (2 L MI-ACB) and single-layer multi-inductor active cell balancing with a state of charge-based controller. In the MATLAB/SimScape environment, the inductor-based balancing method for 52 V battery systems is implemented based on the comparison, and the results are explained. The model is tested with OPAL-RT 5700 real-time HIL Simulator and compared with simulation results to show its effectiveness.

## Introduction

The automotive sectors are currently developing lightweight technology, cognitive ability, communication, and electrification. Due to their near-zero emissions and energy efficiency, electric cars, or EVs, have seen substantial development to keep up with this anticipated trend. Since lithium-ion batteries (LIBs) have long been the primary energy source for electric vehicles (EVs), their intrinsic qualities such as their high energy density, extended cycle life, and low self-discharge rate have made LIBs ubiquitous in the field. A LIB pack is required to meet the high voltage requirements of EVs because a single battery cannot provide the voltage needed for an actual EV to operate. Multiple batteries must be connected in series. Nevertheless, due to variations in production techniques and application settings, there exists an inconsistency in the battery pack that significantly restricts its capacity, power, and longevity and may potentially result in safety violations^1–5^. An adequate active equalisation circuit and accompanying control scheme must be designed to meet the consistency requirements of each battery pack cell and minimise LIB inconsistency. Lithium-ion battery technology is implemented for electric vehicles and spacecraft because of its high usable energy, prolonged life cycle, battery safety, and low self-discharge. A lithium battery pack needs an efficient battery management system (BMS) to monitor the individual cell voltage, current, temperature, state of charge, and discharge. The capacity of the battery pack is achieved by connecting cells in series and parallel based on mPnS theory. A module is formed by connecting m number of cells in parallel to get the Ampere-Hour (Ah) capacity of the battery pack and n number of modules are connected in series to get the rated voltage of the battery pack^6^. The cells have unbalanced voltages due to their internal impedances, which adversely affect the battery pack’s performance when it charges and discharges. Hence, an efficient cell balancing technique is required to maintain the cell’s voltages in a balanced condition^7,8^. Cell balancing is done by balancing either the state of charge or voltage of the cells.

**Fig. 1:**
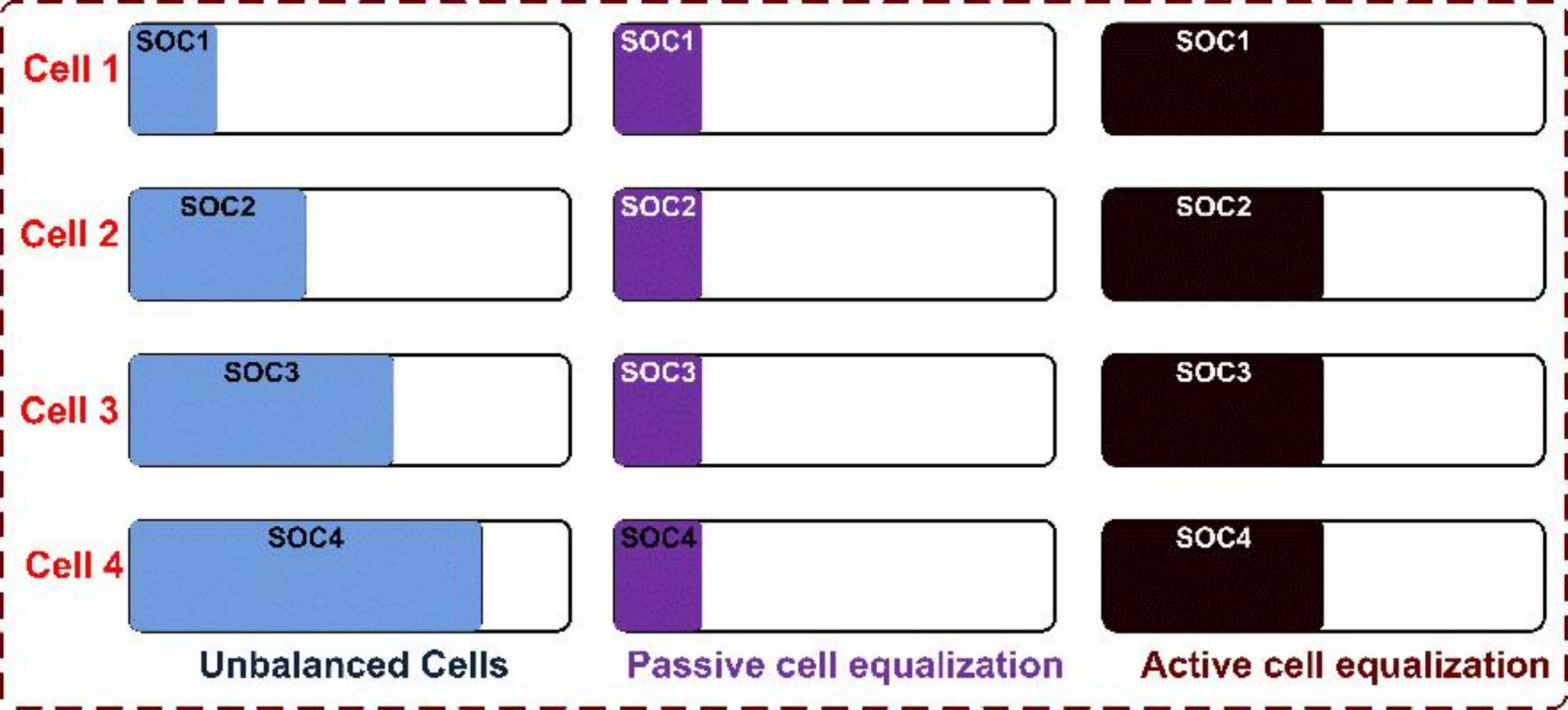
The basic concept of cell balancing.

**Fig. 2:**
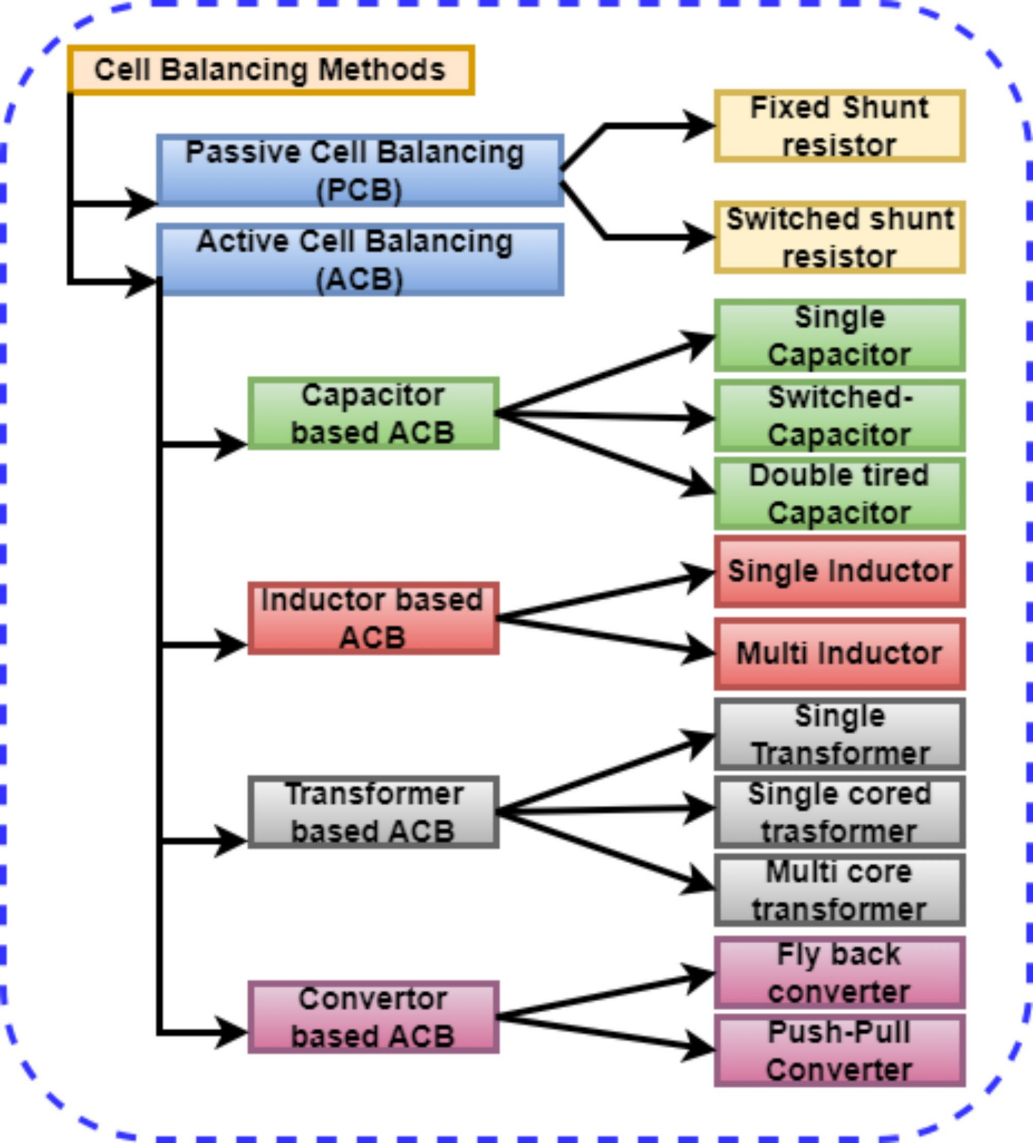
Classification of cell balancing topologies.

Cell balancing is an important factor in a large battery bank. The imbalance in the cells adversely affects the health of the battery pack (9–11). The overall efficiency of the battery pack is affected by the weakest cell in the string when it is discharged below a specified limit, leading to thermal runaway, and causing safety issues (12–14). The highest SOC cells share energy with the lowest SOC cells to balance the string based on the control algorithm, as shown in Fig. 1. The active cell balancing method overcomes the drawbacks of the passive cell balancing method. Figure 2 shows the classification of cell-balancing methods based on the SOCs of the cells.

According to^14^, the passive balancing technique is widely employed in electric vehicle applications since it is inexpensive and simple to install. Low efficiency, a lengthy balancing period, and excessive heat dissipation from the balancing resistor are the main causes of the issue. A low balancing current is necessary to control thermal problems and lessen power loss^15^. To manage power dissipation as efficiently as possible^16^, recently implemented MOSFET (metal oxide semiconductor field-effect transistor) switches as variable resistors, replacing traditional balancing resistors. To address significant power loss and regulate any unintentional temperature rise that causes pack degradation, the passive scheme must incorporate a suitable thermal management system. According to^17^, active topologies are currently taking the place of conventional passive systems to address power loss and heat dissipation problems.

**Fig. 3:**
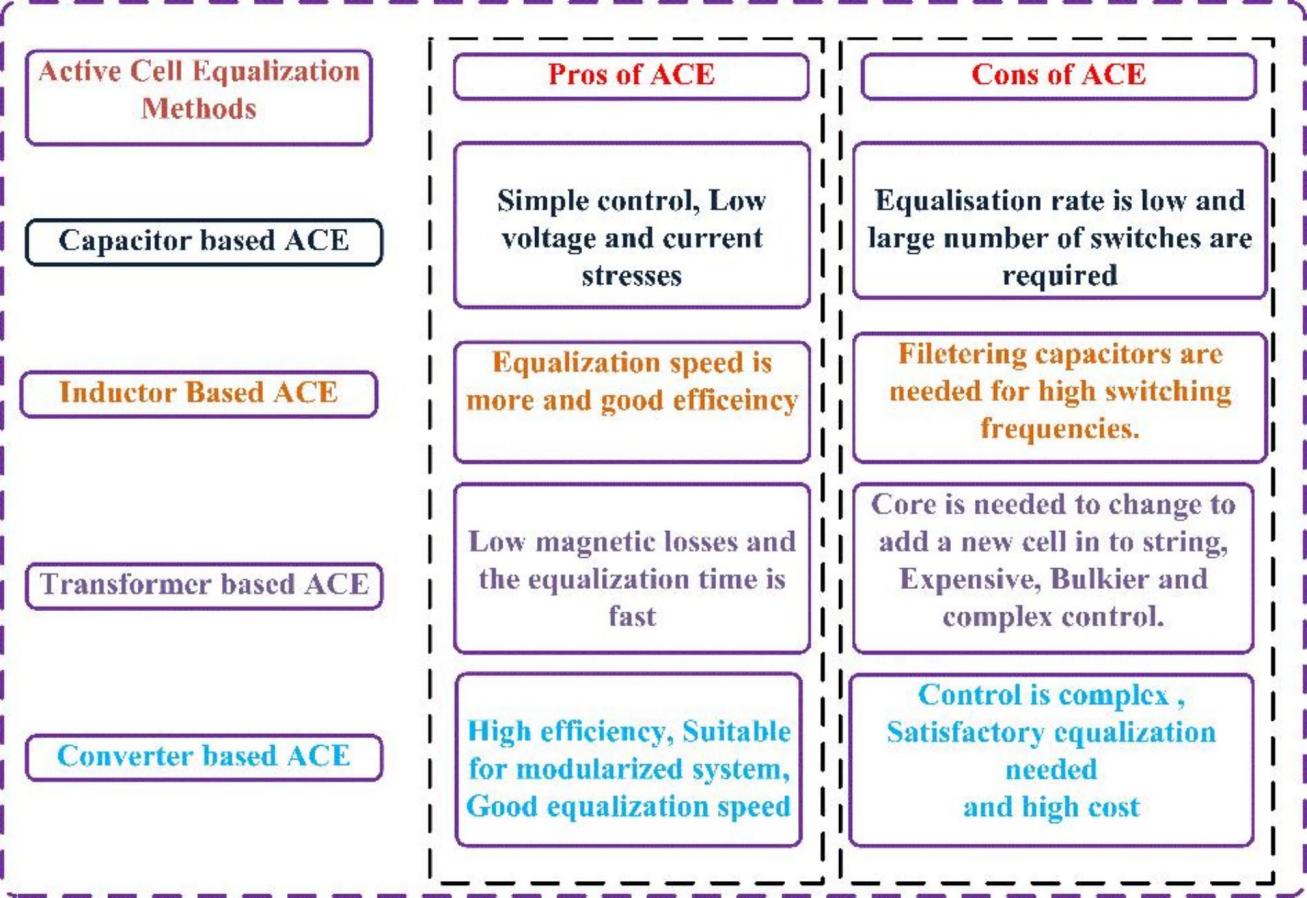
Pros and Cons of ACE.

In Active Cell Balancing/Equalisation (ACE), the cells with higher and lower SOCs mutually share the energy for cell balancing (19–21). The classification of ACE is done based on energy-storable elements such as inductors^21^, capacitors^22^, multi-winding transformers^23^, and DC-DC converters^24^. According to^25^, the capacitor-based approach has a low implementation cost but a slow balancing speed. A transformer is required for a transformer-based scheme, resulting in a fast-balancing speed but also a high cost^26^. The inductor approach performs better when it balances speed and implementation cost^27^. In real-world situations, a greater number of batteries are packed together to generate greater DC voltages. In these situations, it is challenging to design a multi-winding transformer or handle an enormous quantity of DC-DC converters. Figure 3 illustrates the advantages and disadvantages of active cell balancing.

The inductor based ACB method utilizes an inductor for energy storage. By regulating the charging and discharging operations of the inductor, energy may be transferred from a battery with a higher state of charge (SOC) to one with a lower SOC^28,29^. The duration and efficacy of the balancing process are significantly influenced by the charging and discharging cycles of the inductor. The BMS assesses the disparity in SOC values between the battery with a higher SOC and the battery with a lower SOC^30^. Subsequently, the inductor based ACB commences operation to diminish this differential value^31,32^.

In the ACB that is based on capacitors, capacitors are used to transport energy from the more powerful battery to the less powerful battery. Switches are used to swap the capacitors to accomplish this function. The capacitor is charged by the battery that has a higher state of charge (SOC), and then it discharges when it is transferred to the battery that has a lower SOC value^33,34^. The process of alternating current battery (ACB) is also known as the charge-shuttling process. This is because the process includes the transfer of charge between the cells, and the control operation is simple^35^. On the other hand, this approach has some drawbacks, including the fact that it causes a loss of energy during the process of charging and discharging the capacitors, switching losses between the switches, and a large amount of time is necessary for charging and discharging the capacitors^36,37^.

The equalization of the cells in the DC/DC converter-type ACB is achieved using DC/DC converters. The circuit governing the converter switches for executing the cell balancing operation renders this ACB procedure relatively intricate^38–42^. Nevertheless, the equalization duration required by the battery pack cells is relatively shorter in comparison to the capacitor based ACB. The DC/DC converter method is often categorized as quasi-resonant, Cuk, ramp, flyback, buck-boost, and full-bridge converter types ACB^43^.

In the transformer based ACB, current is directed into the transformer’s main winding from a more powerful cell or cells. The main winding generates current in the secondary winding, which is connected to one or more cells. The transformer facilitates the equalization of the cell characteristics^41^. This method offers more efficiency than the inductor based ACB^42^. This procedure, however, entails higher costs relative to other kinds due to the inclusion of the transformer and intricate circuitry^43^.

Transitioning from cell balancing techniques to state of charge (SOC) estimation highlights the importance of accurate battery management in enhancing the performance and longevity of energy storage systems. Effective cell balancing ensures uniform energy distribution among battery cells, mitigating the effects of capacity fading and thermal runaway. However, to fully optimize battery usage, precise SOC estimation becomes critical. This estimation provides insights into the battery’s remaining charge, directly influencing power management strategies in applications such as electric vehicles (EVs) and renewable energy systems.

Several SOC estimation techniques have been developed, each with its advantages and challenges. Traditional methods, such as the coulomb counting methods^44,45^, Kalman filter^45,46^and extended Kalman filter^47,48^, adaptive extended Kalman filter^49,50^, particle filter^51^, have been widely used due to their ability to incorporate real-time measurements and adapt to dynamic changes. More recently, machine learning approaches^52–56^, Neural network based approaches^76,77^and online estimation approaches^57–60^ have gained traction, leveraging large datasets to improve accuracy and robustness in various operating conditions in artificial intelligence and deep learning models, the particle filter algorithm remains a strong contender for SOC estimation. Its strength lies in its ability to handle nonlinear and non-Gaussian systems effectively, making it well-suited for battery dynamics that can be complex and variable.

The particle filter is by maintaining a set of particles, each representing a possible state of the system, and updating these particles based on the likelihood of observed measurements. This approach allows for better handling of uncertainties and provides more robust estimation in scenarios where battery behaviour is influenced by various factors, such as temperature variations and load changes. Thus, even in the presence of more advanced techniques, the particle filter remains relevant for SOC estimation due to its robustness and flexibility in managing uncertainty, making it a suitable choice for my research on battery management systems.

As an alternative to the equalisation techniques, the inductive equalisation circuit features simple control of the equalisation current, an easy-to-implement control concept, and an intermediate energy storage element in the form of inductance for energy transmission. Thus, in the last few years, it has progressively emerged as a hotspot in the study of active equalisation techniques. A proposed active equalisation circuit in Ref^61^. utilizes inductance to provide uniformity within the battery pack; its control principle is straightforward and easy to apply, parallel to a switched capacitor equalisation circuit. Ref^62^. describes a single-layer inductor active equalisation circuit that controls an on-off switch to facilitate energy transfer between two adjacent batteries, so efficiently improving the disparities between different battery packs. Ref^63^. suggested a class of centralised active balancing circuits based on numerous switches where the switch matrix was used to determine the necessary balancing cells for balancing. The energy transfer within the battery pack is the only thing that these equalisation circuits can accomplish, which could significantly lengthen the equalisation time. A connected inductance equalisation circuit was described in Ref^64^. to reduce the equalisation time. Nevertheless, the circuit is unable to realise the energy transfer between the battery packs. Furthermore, it only worked in situations where the number of cells was equal.

To summarize, Major active balancing topologies include:

(1) Capacitor-based, known for its simplicity and reliability. This method requires a voltage difference for balance, making it unsuitable for wide-voltage battery packs.

(2) Transformer-based balancing topology offers simplified switching and faster balancing. It has drawbacks such as expensive circuit costs and big circuit sizes.

(3) Converter-based balancing topology offers great integration and excellent balancing performance. However, it has drawbacks such as complex design and expensive application costs.

(4) Inductor-based balancing topology offers excellent efficiency and rapid speed. Inductor balancing transfers energy as current, making it ideal for situations with tiny voltage differences between batteries and a wide voltage platform.

However, it has drawbacks such as complex control and numerous switches^30,65^. Due to the low voltage changes between cells and the wide voltage platform of retired power batteries, this article focuses on an inductor-based balancing topology structure. The proposed inductor-based balancing architecture in ref^66^has issues with repetitive charging and discharging, reducing its efficiency^66^. This research suggests a faster and more efficient balancing approach to reduce energy loss.

The following are the contributions of this work:

1. Introduced Two-layer (2 L) and Single-layer (1 L) Multi-Inductor Active Cell Balancing (MI-ACB) topologies to improve Four LIB battery pack SOC balancing efficiency and speed.
2. The Two Layer MI-ACB topology balanced all cells in 54 s in simulation and 65 s in real-time hardware-in-the-loop (HIL) testing, achieving 42% SOC and 99.974% efficiency.
3. Provided a cost-effective Single layer MI-ACB topology with 99.99% efficiency 108 s simulation and 110 s HIL cell balancing.
4. Combined a multi-layer inductor-based structure with a dependable SOC-based control algorithm to reduce balancing time.
5. Future work to tune inductance, resistance, and switching frequency to improve efficiency and reduce balancing time, and to experimentally test the control system considering temperature and age propagation.

The paper is organised into the following sections: the proposed methodologies are discussed in Sect. 2. The SOC estimation based on the PF algorithm is discussed in Sect. 3. The Battery Model, Parameter Identification, and PF Algorithm Implementation are discussed in Sect. 4. The control strategy for the proposed inductor based active equalisation circuit is discussed in Sect. 5. The cell balancing efficiency is reported in Sect. 6. The simulation results and discussion are reported in Sect. 7. The Hardware results and discussion are reported in Sect. 8. The discussion and comparison of the hardware-tested results are presented in Sect. 9. Lastly, the conclusion is presented in Sect. 10.

## Methodology

### Two-layer active equalisation topology

**Fig. 4:**
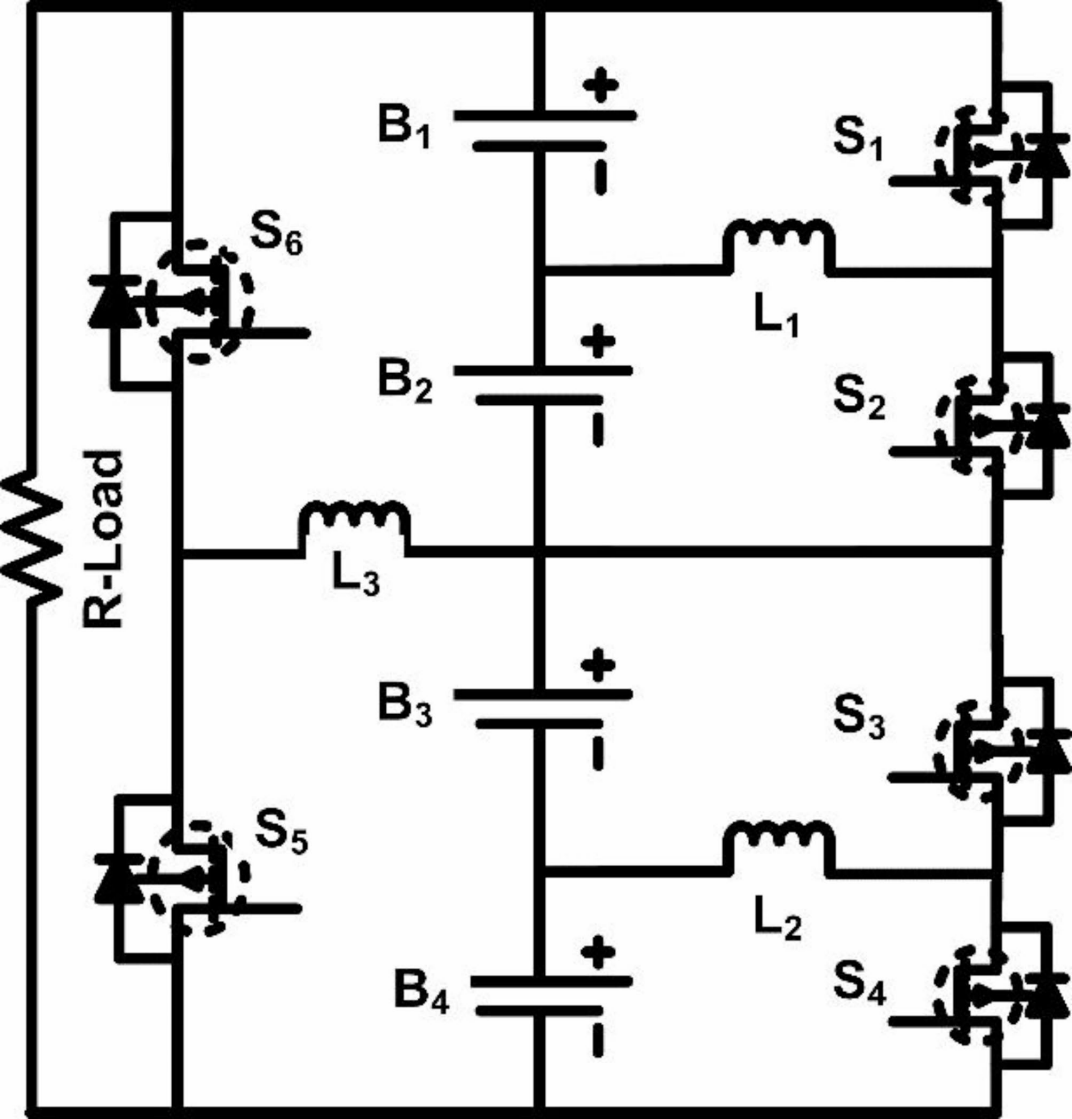
Two-layer inductor based active cell equalisation topology.

In the proposed battery balancing circuit, a two-layer structure is used to efficiently transfer energy among cells in a series-connected lithium-ion battery pack. This layered approach enables faster balancing by allowing energy exchange between adjacent cells and subsequently across larger cell groups. The two-layer model is depicted in Fig. 4.

To demonstrate the functionality of the proposed two-layer active equalization topology, the following operating cases are presented. Each case represents a different SOC configuration among the four series-connected cells and illustrates how the system performs cell balancing in two stages: first-layer balancing between individual cells within pairs, followed by second-layer balancing between the two paired groups. The operating cases are as follows:

#### Case 1

SOC_1_ > SOC_2_ > SOC_3_ > SOC_4_.

In this scenario, the SOC of each cell decreases from B_1_ through B_4_, with SOC_1_ being the highest and SOC_4_ the lowest. The two-layer equalization process begins with the first layer, where balancing occurs within each pair (B_1_ and B_2_, as well as B_3_ and B_4_). Since SOC_1_ is higher than SOC_2_, Switch S_1_ is activated to allow energy transfer from B_1_ to B_2_ through inductor L_1_. This inductor stores energy while S_1_ is on and discharges it to B_2_ when the switch turns off, balancing their SOC. Concurrently, Switch S_3_ is activated to facilitate energy transfer from B_3_ to B_4_ via inductor L_2_, equalizing the SOC levels between B_3_ and B_4_. After these first-layer balancing actions, B_1_ and B_2_ achieve the same SOC, as do B_3_ and B_4_, but the SOC of group B_1_-B_2_ remains higher than that of group B_3_-B_4_. The second layer is then engaged, where Switch S_6_ turns on, activating inductor L_3_ to transfer energy from the higher SOC group (B_1_-B_2_) to the lower SOC group (B_3_-B_4_). This step completes the balancing process across all cells, ensuring an even distribution of charge.

#### Case 2

SOC_2_ > SOC_3_ > SOC_4_ > SOC_1_.

In this case, the highest SOC is found in B_2_, followed by B_3_, then B_4_, and finally B_1_. The first-layer balancing process addresses this order by activating Switch S_2_ to transfer energy from B_2_ to B_1_, using inductor L_1_. When S_2_ is on, L_1_ stores energy from B_2_, which is then discharged to B_1_, effectively equalizing their SOC levels. Simultaneously, Switch S_3_ is activated to transfer energy from B_3_ to B_4_ via inductor L_2_, balancing the SOC levels between these two cells. Once the first-layer balancing is complete, pairs B_2_-B_1_ and B_3_-B_4_ have equal SOC within each pair. However, since the SOC of group B_2_-B_1_ is higher than that of group B_4_-B_3_, the second layer is engaged. Switch S_5_ is turned on, allowing L_3_ to transfer energy between the two groups, ensuring a balanced SOC across the entire series of cells.

#### Case 3

SOC_4_ > SOC_3_ > SOC_1_ > SOC_2_.

In this configuration, SOC_4_ is the highest, followed by SOC_3_, SOC_2_, and then SOC_1_. The first-layer balancing addresses this arrangement by activating Switch S_4_ to enable energy transfer from B_4_ to B_3_ through inductor L_2_. As S_4_ turns on, L_2_ stores energy from B_4_ and releases it to B_3_, equalizing their SOC levels. Meanwhile, Switch S_2_ is activated to transfer energy from B_2_ to B_1_ through inductor L_1_. This step balances the SOC between B_2_ and B_1_. After first-layer balancing, the SOC levels of B_4_-B_3_ and B_2_-B_1_ are equal within each pair, but group B_4_-B_3_ has a higher SOC than group B_2_-B_1_. To address this imbalance, the second layer is activated, where Switch S_5_ turns on, allowing inductor L_3_ to facilitate energy transfer from B_4_-B_3_ to B_2_-B_1_, completing the balancing process for all cells in the pack.

#### Case 4

SOC_3_ > SOC_4_ > SOC_1_ > SOC_2_.

**Fig. 5:**
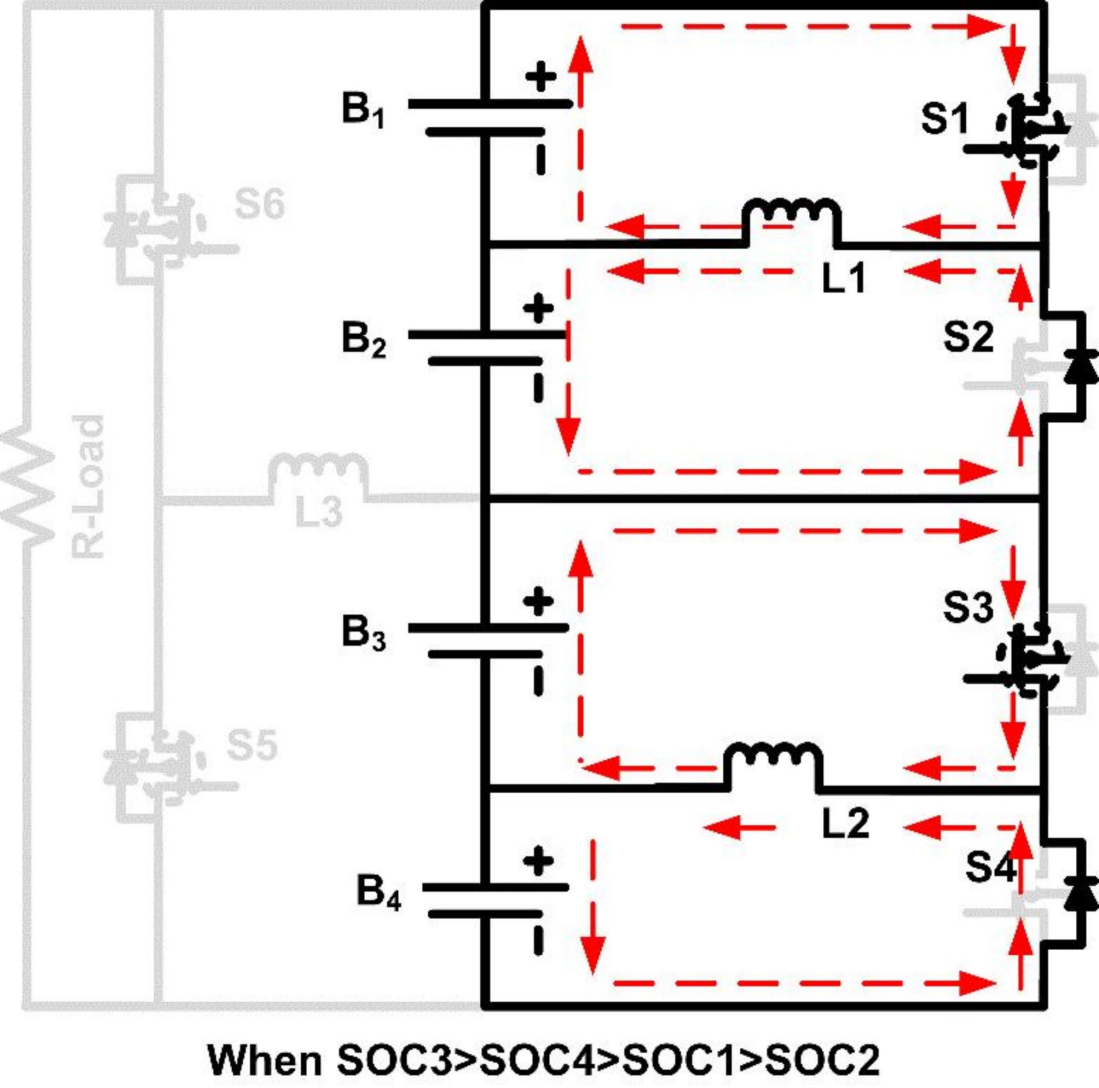
Operating mode when SOC_3_ > SOC_4_ and SOC_1_ > SOC_2_.

**Fig. 6:**
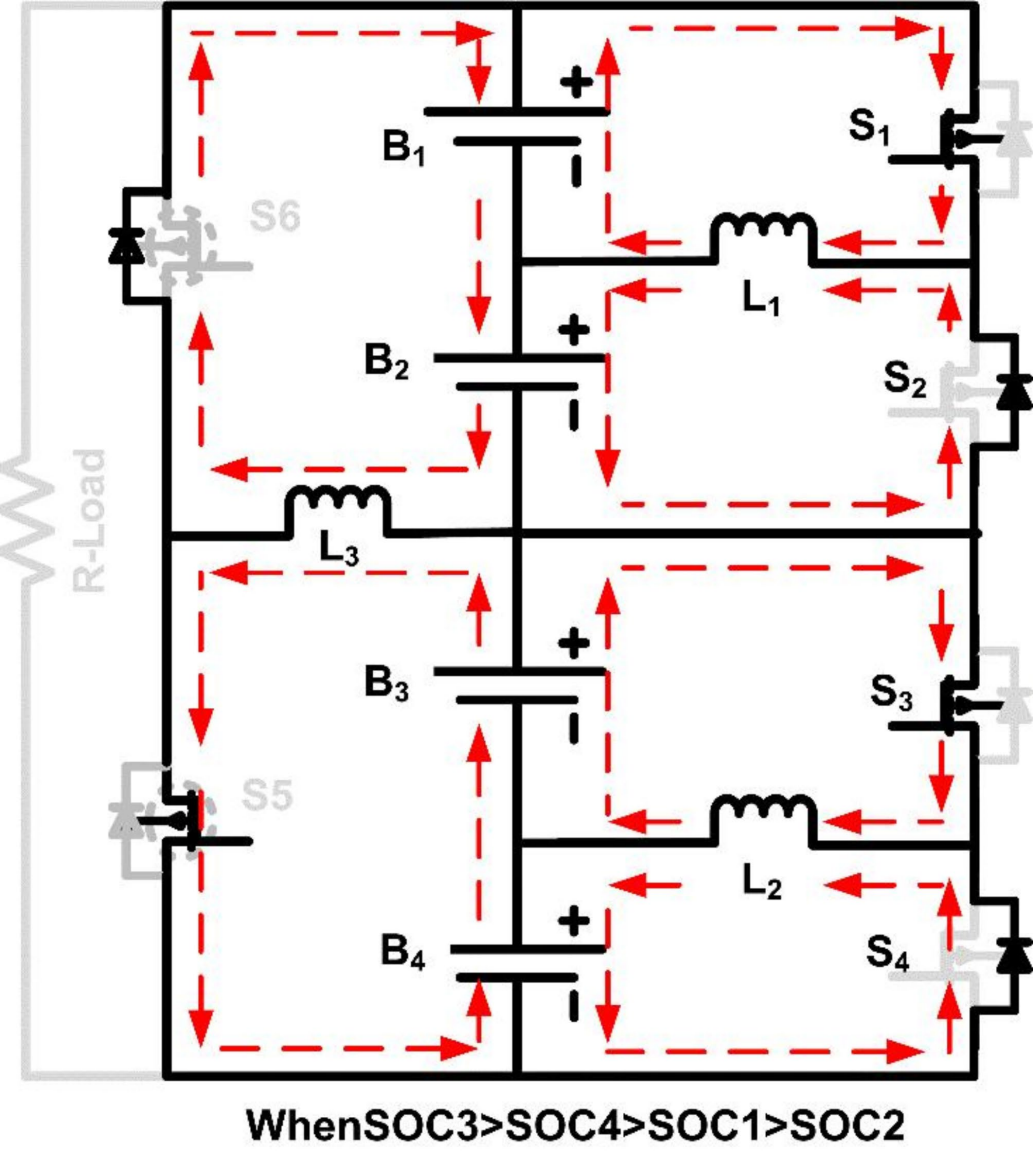
Operating mode when SOC of B_1_ > B_2_, B_3_ > B_4_ and B_3_B_4_ > B_1_B_2_.

In this scenario, the SOC is highest in B_3_, followed by B_4_, then B_1_, and finally B_2_. The first-layer balancing process starts by activating Switch S_3_, which allows energy transfer from B_3_ to B_4_ via inductor L_2_. As S_3_ turns on, L_2_ stores energy from B_3_ and discharges it to B_4_, balancing their SOC. Simultaneously, Switch S_1_ is activated to transfer energy from B_1_ to B_2_ through inductor L_1_, equalizing the SOC levels of these cells. After completing the first-layer balancing, B_3_ and B_4_ have equal SOC, as do B_1_ and B_2_. However, the SOC of group B_3_-B_4_ remains higher than that of group B_1_-B_2_, so the second layer is engaged. Switch S_6_ is turned on, activating inductor L_3_ to transfer energy from the higher SOC group (B_3_-B_4_) to the lower SOC group (B_1_-B_2_), achieving full balance across all cells. Figures 5 and 6 illustrate the operational modes of cell balancing for case 4. This topology reduces the time needed for balancing and optimizes energy transfer, as energy is first equalized within pairs, and then between groups. By designing the system in this modular, multi-layered fashion, the balancing circuit remains effective and scalable, potentially accommodating larger packs with minimal adjustments to the core design.

The two-layer structure enables each cell pair to be balanced independently at the first level before balancing occurs between paired groups. This modular design can scale up to multiple layers, depending on the total number of cells.

For a system with M cells in series, the number of required layers (l_N_), switches (S_N_), and inductors (L_N_) can be calculated as follows:

The number of layers (l_N_) is calculated from the below equation:

1 $$\:{l}_{N}={log}_{2}\left(M\right)$$

The number of Switches (S_N_) is calculated from the below equation:

2 $$\:{S}_{N}=\sum\:_{P=0}^{l}\left(\frac{M}{{2}^{P}}\right)$$

The number of inductors (L_N_) is calculated from the below equation:

3 $$\:{L}_{N}=\sum\:_{P=1}^{l}\left(\frac{M}{{2}^{P}}\right)$$

### Optimised single-layer active equalisation topology

**Fig. 7:**
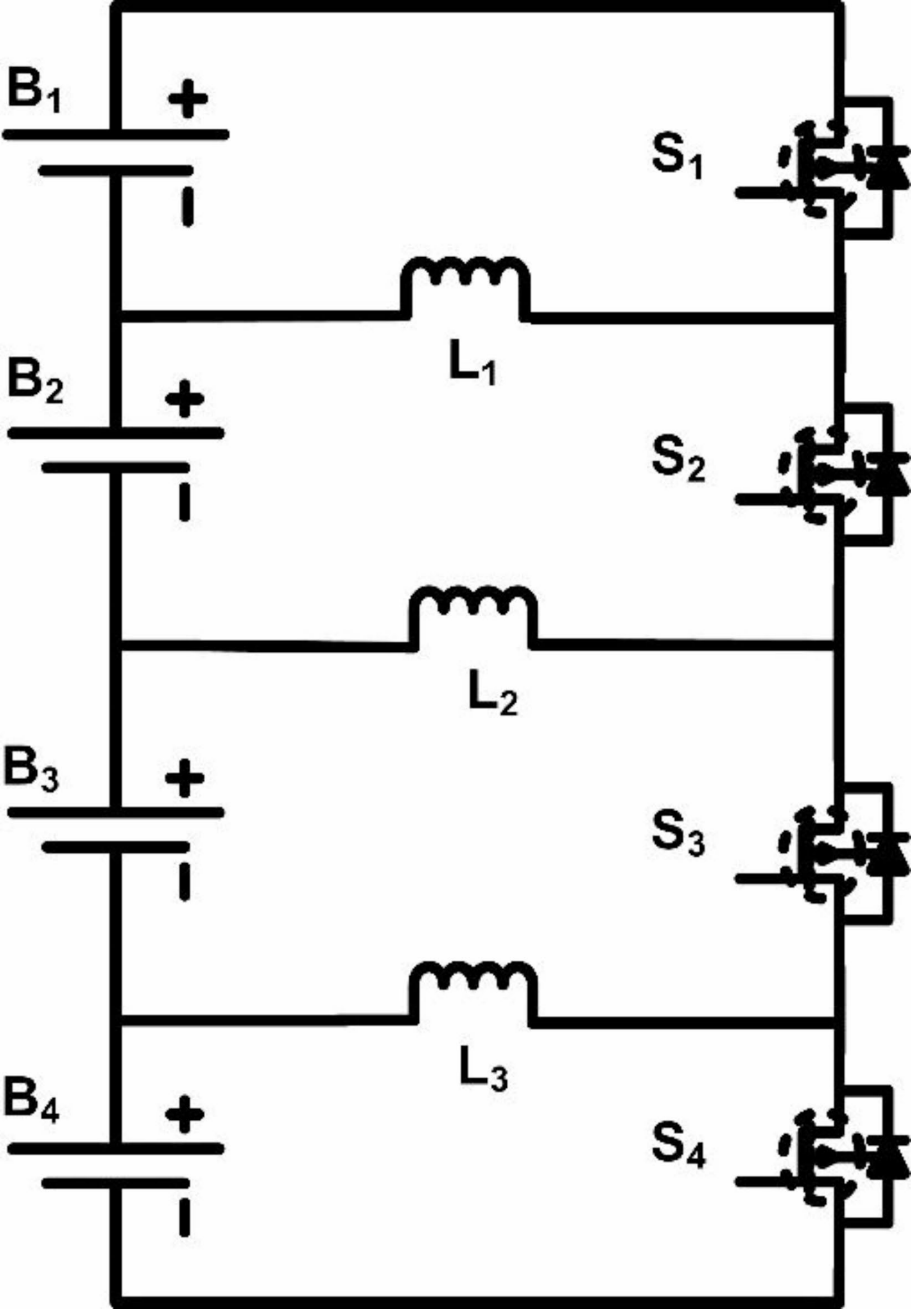
Optimized single-layer inductor based active cell equalisation topology.

The optimized single-layer cell balancing model employs a streamlined, single-layer circuit design that minimizes component requirements while effectively balancing the State of Charge (SOC) across multiple cells. In this topology, three inductors (L_1_, L_2_, L_3_) and four switches (S_1_, S_2_, S_3_, S_4_) are configured to handle energy transfer between cells based on their SOC values. The simplicity of this structure enhances efficiency by reducing switch count and system complexity, making it well-suited for compact and efficient battery management systems. The proposed optimized single-layer cell balancing model is shown in Fig. 7.

The number of switches is equal to the number of cells, given from the below equation:

4 $$\:{S}_{N}=M$$

The number of inductors (L_N_) is calculated from the below equation:

5 $$\:{L}_{N}=M-1$$

where M is the total number of cells in the pack. These elements facilitate controlled energy transfer between cells, managed through a logic-based control system that adjusts switching actions depending on the SOC differences between adjacent cells.

The controller continuously monitors each cell’s SOC and executes specific switching actions based on predetermined SOC thresholds. The algorithm operates as follows:

#### Case 1

SOC_1_ > SOC_2_ > SOC_3_ > SOC_4_.

Cell B_1_, having the highest SOC, transfers energy to B_2_ by activating switch S_1_. When S_1_ is activated, inductor L_1_ stores energy from B_1_ and subsequently releases it to B_2_. Simultaneously, B_3_, which also has a higher SOC than B_4_, shares its energy with B_4_ through switch S_3_ and the inductor L_3_.

Once the SOCs equalize between B_1_-B_2_ and B_3_-B_4_, the intermediate cells, B_1_ and B_2_, collectively transfer energy to B_3_ and B_4_ by engaging switches S_1_ and S_2_ via inductor L_2_.

#### Case 2

SOC_2_ > SOC_3_ > SOC_4_ > SOC_1_.

Cell B_2_, now the highest, discharges energy to B_1_ through S_2_ and L_1_, while B_3_ continues to transfer energy to B_4_ through S_3_ and L_3_. When SOCs between B_1_-B_2_ and B_3_-B_4_ are balanced, switches S_3_ and S_4_ are activated to transfer energy across B_3_-B_4_ and B_1_-B_2_ pairs through inductor L_2_.

#### Case 3

SOC_3_ > SOC_4_ > SOC_1_ > SOC_2_.

In this case, B_2_ transfers energy to B_1_ through S_2_ and L_1_, while B_4_ balances with B_3_ via S_4_ and L_3_. Once B_1_-B_2_ and B_3_-B_4_ are balanced, further energy balancing between the pairs occurs through switches S_3_ and S_4_ using inductor L_2_.

#### Case 4

SOC_4_ > SOC_3_ > SOC_1_ > SOC_2_.

Cell B_1_, having a higher SOC than B_2_, transfers energy to B_2_ via S_1_ and L_1_, while B_3_ continues to balance with B_4_ through S_3_ and L_3_.

After balancing between B_1_-B_2_ and B_3_-B_4_, further energy transfer is performed between B_4_-B_3_ and B_2_-B_1_ pairs by activating switches S_3_ and S_4_ through inductor L_2_.

The different SOC-based switching continuously monitors the SOC of the cells in the battery pack. Through these SOC-based switch activations, the model balances each cell’s charge by ensuring that only specific pairs transfer energy at any given time, minimizing simultaneous switch activations and reducing potential power loss. The continuous balancing process could lead to significant power loss through the control circuit and component resistance. To mitigate this, a threshold SOC value is established. The controller initiates balancing only when SOC differences exceed this threshold, thus reducing unnecessary switching cycles and associated energy losses.

**Fig. 8:**
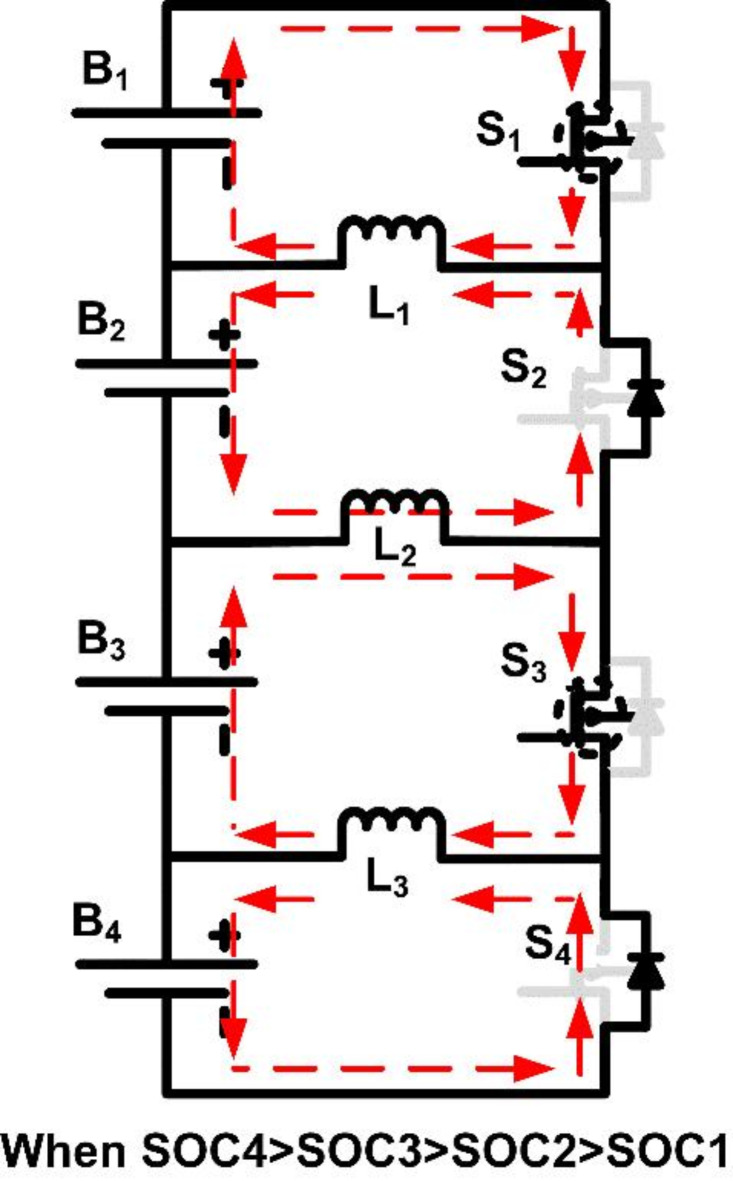
Operating mode when SOC1 > SOC2 and SOC3 > SOC4.

**Fig. 9:**
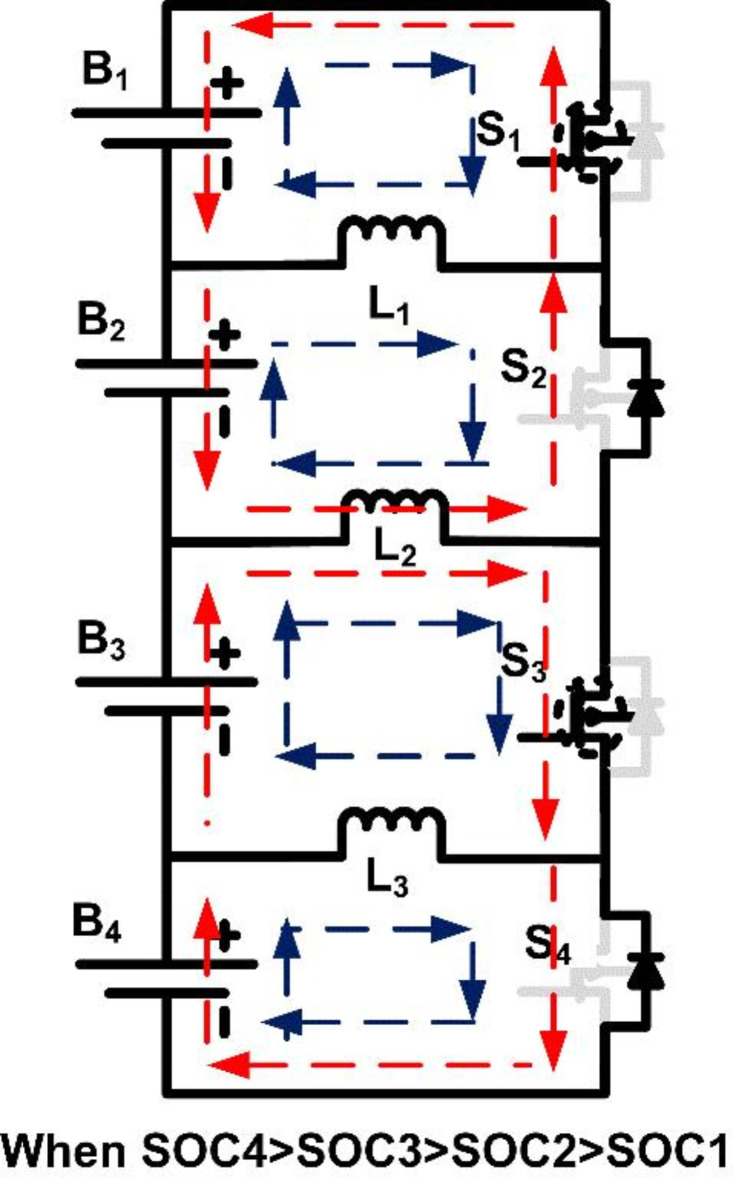
Operating mode when SOC of B3B4 > B1B2.

Figures 8 and 9 illustrate the operational modes of cell balancing for case 1. These figures depict various switching scenarios and inductor roles in energy transfer, demonstrating the dynamic responses of the system to varying SOC conditions.

## Battery model, parameter identification, and PF algorithm implementation

### Battery model and parameter identification

In this work, a 2RC equivalent circuit model was chosen for modelling lithium-ion batteries due to its accuracy and computational efficiency. This model consists of two resistor-capacitor (RC) branches, which effectively capture the battery’s dynamic behaviour, including voltage hysteresis and transient response. The parameters of this model such as internal resistance, charge transfer resistance, and double-layer capacitance were determined through Hybrid Pulse Power Characteristic (HPPC) test data. This data-driven approach allows the model to reflect real-world performance under diverse operational conditions, which is crucial for reliable SOC estimation in active balancing applications. This choice ensures an optimized trade-off between model simplicity and accuracy in capturing essential electrical characteristics.

### State-of-charge (SOC) estimation using PF algorithm

The Particle Filter (PF) algorithm was used to enhance SOC estimation accuracy in our balancing system. The PF algorithm is a recursive Bayesian filter, which is particularly robust in managing the non-linearities and noise found in battery behavior, offering improved SOC estimation over traditional methods. By applying this filter to the 2RC model, our approach corrects estimation errors that might arise from direct Coulomb counting, such as cumulative integration drift and inaccuracies at lower SOCs^62,67–69^. To implement PF in our work, the following steps were taken:

### Initialization

The PF algorithm begins by defining an initial probability density function (PDF) for the SOC, which represents the likely distribution of SOC values for each battery cell based on prior knowledge or initial measurements.

A subset of particles (representing possible SOC values) is then drawn from this initial distribution. Each particle has an assigned weight representing its likelihood of being close to the true SOC.

### Prediction and update of dynamic parameters

For each time step, dynamic parameters such as current, voltage, and temperature are predicted based on the battery model (2RC model). Each particle’s SOC is updated according to the battery’s behaviour and model predictions. This step incorporates the effects of charge/discharge cycles and compensates for any transient battery behaviour.

### Weighting and resampling

After predicting the SOC, the algorithm compares the predicted state with the observed measurements. This is where measurement noise and uncertainties are managed. Each particle’s weight is adjusted based on how closely its predicted SOC aligns with the measured SOC, emphasizing particles closer to the measured value.

A resampling step follows, where particles with lower weights are replaced by copies of particles with higher weights. This process helps prevent the filter from diverging and reduces cumulative errors in SOC estimation.

### Estimation output

The SOC estimate is derived as the weighted mean of all particle SOC values. This approach smooths out individual measurement noise and improves accuracy.

### Threshold control in balancing

In the balancing strategy, balancing switches are activated when the difference in SOC between cells (SOC_i_ - SOC_n_) is greater than 1% and are turned off when the difference is reduced to below 0.01%. Accurate SOC estimation using PF ensures that the balancing system triggers precisely, preventing unnecessary balancing cycles and minimizing energy loss.

**Fig. 10:**
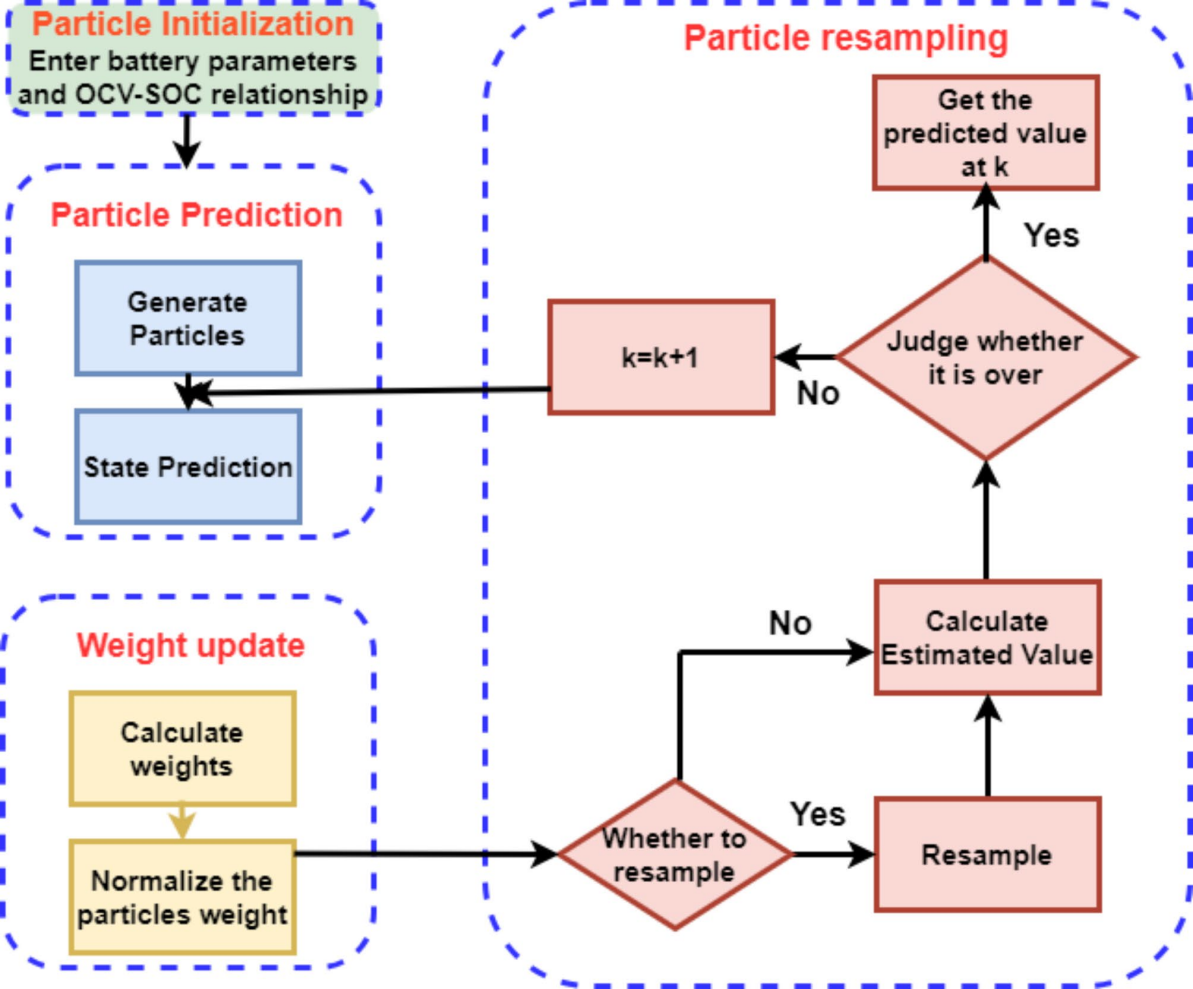
Structural diagram of PF.

Figure 10. illustrates the structural diagram for this process^70^. As illustrated in Fig. 10, this technique uses the probability density function to estimate the sample mean value after randomly selecting a subset of particles for initialization from the initial density distribution function. Next, the dynamic parameters are anticipated, and each particle’s weight is updated based on the estimated outcome.

**Fig. 11:**
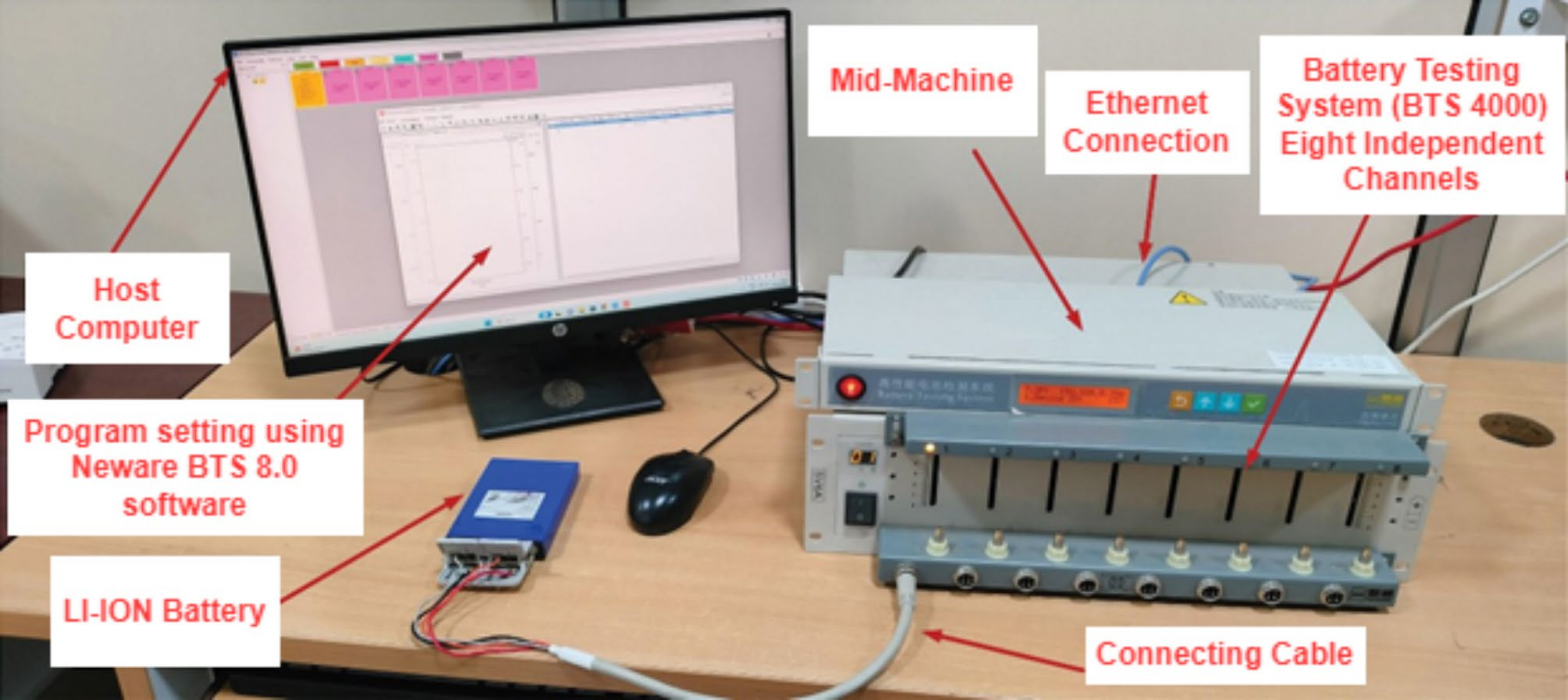
Testing platform using BTS 4000 Battery testing system.

**Fig. 12:**
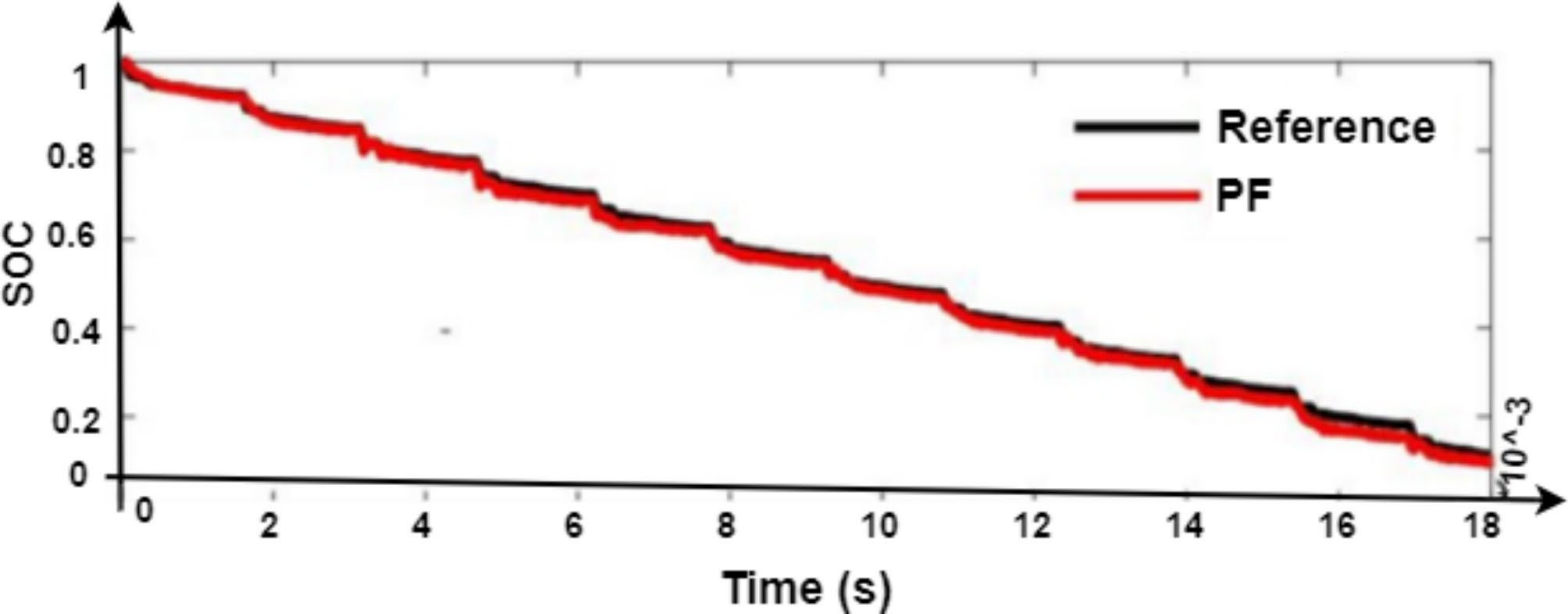
Battery SOC estimation curve based on PF algorithm.

Lithium-Ion batteries are evaluated using the BTS 4000 battery testing system shown in Fig. 11 to further evaluate the viability of the PF-based SOC estimate in this work. It is important to note that hybrid pulse power characteristic (HPPC) test data is used to determine the parameters of the battery model. Figure 12 displays the SOC estimation curve and its inaccuracy based on the PF algorithm.

## Comparative analysis of PF Algorithm vs. Coulomb counting for SOC estimation

For validation, a comparative analysis between the PF algorithm and Coulomb counting was performed using our dataset, encompassing four cells under varying conditions. The analysis demonstrated that:

PF provided consistently accurate SOC estimation by reducing cumulative errors often seen in Coulomb counting. PF corrected SOC estimation drift, maintaining accuracy within ± 1% of true SOC, while Coulomb counted accumulated errors over time. Under dynamic charge-discharge conditions, PF adapted to fluctuations more effectively than Coulomb counting, showcasing its robustness in real-time applications.

By integrating PF with the 2RC model, we achieved improved SOC estimation accuracy, critical for reliable balancing control and battery longevity. This novel approach justifies our claim of enhanced accuracy in SOC calculation, demonstrating its value in optimizing battery pack performance and minimizing cell mismatch during balancing operations. Table 1 shows the comparison between SOC estimation between PF and Coloumb counting techniques. Average SOC Error Indicates the average error between estimated SOC and actual SOC over multiple simulations. PF shows a lower average error due to its probabilistic correction capabilities. Maximum SOC Error reflects the highest deviation observed in each method. PF’s maximum error remains within ± 2.5%, while CC’s error occasionally spikes to ± 5%. Overextended cycles, cumulative error (drift) is significantly lower in PF, which corrects SOC values at each update cycle, compared to the uncorrected integration error in CC.

**Table 1:** Comparison of SOC estimation methods.

SOC Estimation Method	Average SOC Error (%)	Maximum SOC Error (%)	Cumulative SOC Drift over 500 Cycles (%)
Particle Filter (PF)	± 1.5	± 2.5	± 1.5
Coulomb Counting (CC)	± 3.5	± 5.0	± 4.5

### Control strategy for proposed two-layer and optimized single-layer inductive active equalisation topologies

The opening and closing of the MOSFET corresponding to the battery with a higher state of charge (SOC) is controlled by the multi-layer inductance active balance control technique, which also transfers energy from a higher SOC battery to a lower SOC battery. Consequently, it is possible to determine the relationship between the switch’s on/off state and the transfer of energy. For instance, S_1_ is closed when SOC_1_ > SOC_2_. This zero-state response equation describes the charging loop in Fig. 4, which is a first-order RL circuit:

6 $$\:{V}_{1}={R}_{on}{i}_{l}+L\frac{di}{dt},\:t=0\:\underrightarrow{.}\:{t}_{on}$$

where PWM is the control signal of S_1_, R_on_ is the circuit’s total resistance, and V_1_ and V_2_ are the voltages of Cells 1 and 2, respectively. L is the inductor value of L_1_, i_L_ is the current, and t_on_ is the closing time of S_1_ when it is turned on. The general solution to Eq. (4), a first-order differential equation with constant coefficients, is as follows:

7 $$\:{i}_{l}=\:\frac{{V}_{1}}{{R}_{on}}-\frac{{V}_{1}}{{R}_{on}}{e}^{\frac{-t{R}_{on}}{L}}=\frac{{v}_{1}}{{R}_{on}}\left(1-{e}^{-t}\frac{{R}_{on}}{L}\right),\:t=0\:\to\:\:{t}_{on}$$

The following equation applies when the two batteries’ SOC differences reach the beginning threshold:

8 $$\:{SOC}_{1}-{SOC}_{2}\ge\:\varDelta\:{SOC}_{threshold}$$

During this period, the current reaches its maximum value when switch S_1_ is turned on. The magnitude of the current can be calculated from the following equation:

9 $$\:{i}_{max}={i}_{l}=\frac{{V}_{1}}{{R}_{on}}\left(1-{e}^{\left(\frac{{-tR}_{on}}{L}\right)}\right)$$

When PWM is low, S_1_ is switched off. During this process, the magnitude of the current is determined from the following equation:

10 $$\:{i}_{l}=\:{i}_{peak}{e}^{-\left(t-{t}_{on}\right)\frac{{R}_{off}}{L}}-\frac{{V}_{2}+{V}_{D}}{{R}_{off}}\left(1-{e}^{-\left(t-{t}_{on}\right)\frac{{R}_{off}}{L}}\right),\:t={t}_{on}\:\to\:\:{t}_{off}$$

Where V_D11_ is the turn-on voltage of diode D, and R_off_ is the total resistance in the circuit.

**Fig. 13:**
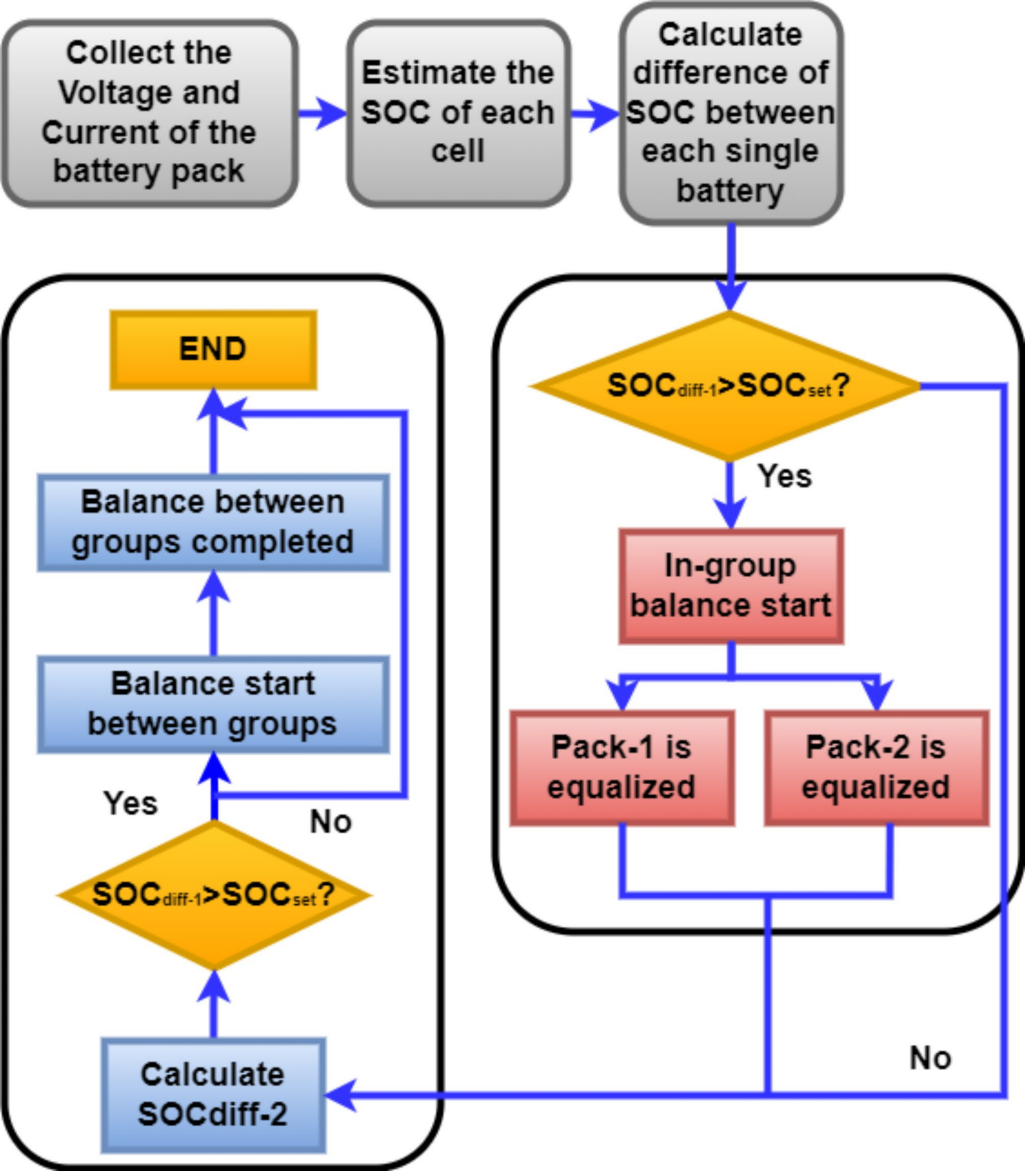
Control logic of Proposed inductance active balancing circuit.

Equations (9) and (10) demonstrate how L_1_ is charged while switch S_1_ is turned off, and how the equalisation current affects switch S_1_’s closing time. The complete equalisation circuit’s balance control technique is created based on this. Figure 13 displays the Proposed inductance active balance control block diagram.

11 $$\:{SOC}_{diff-1}={SOC}_{i}-{soc}_{n}\:\:\:\left(i=\text{1,2},\:.\:.\:.;n=\text{1,2},.\:.\:.;i\ne\:n\:\right)$$

12 $$\:{SOC}_{diff-2}=\:{SOC}_{Pack-1}-\:{SOC}_{Pack-2}$$

Equations (11) and (12) illustrate this. SOC_diff−1_ denotes the SOC difference between two batteries, and SOC_diff−2_ describes the difference between the battery packs. SOC_i_ and SOCn indicate the SOC values of two distinct battery cells, respectively.

**Fig. 14:**
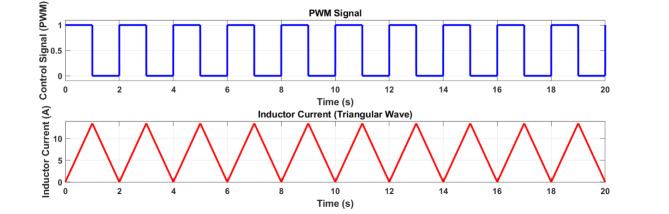
PWM signal and Balancing current of 2 L MI-ACB.

As the principles of balance for the two layers are identical, the balance control approach is verified by looking at the equalisation of the single batteries, Cells 1 and 2. It’s noteworthy that the balance system’s maximum balance current is set at 13.76 A in this instance. We choose the inductance value L to be 10 mH based on the relationship between the maximum current and the inductance value. The MOSFET’s resistance is set to 10mΩ, and the line resistance is disregarded. The equalisation circuit simulation model is constructed in MATLAB/Simulink to assess the rationality of the computed parameters. The following is the circuit’s parameters: The duty cycle is 50% and the control signal is the default Simulink setting for L (10 mH) and R (10 Ω), Fig. 14 displays the change in the control signal and current curve over time.

### Cell balancing efficiency

The current analytical model from^71^, which is derived for active cell balancing topologies based on inductors, is used to compute the charge transfer process’ energy efficiency. Only direct charge transfers between adjacent cells are permitted. The charge transfer process’s energy efficiency(η_c_) is determined by:

13 $$\:{\eta\:}_{C}=1-\frac{{E}_{tpl}}{{E}_{tr}}$$

Where E_tpl_ and E_tr_ indicate the total power loss and transferred energy.

The efficiency is calculated using the Eq. 13. The calculation of energy transfer in the battery pack is as follows:

14 $$\:{E}_{tr}={E}_{{L}_{1}tr}+{E}_{{L}_{2}tr}$$

Where, $\:{E}_{tr}$ indicates total aggregative energy transfer in the battery pack, $\:{E}_{{L}_{1}tr}$ and $\:{E}_{{L}_{2}tr}$ indicates the energy transfer in layer 1 and layer 2.

Energy transfer in layer 1 is calculated as follows:

15 $$\:{E}_{{L}_{1}tr}=\:{{E}_{tr}}_{{B}_{1}{B}_{2}}+\:{{E}_{tr}}_{{B}_{3}{B}_{4}}$$

Where, $\:{{E}_{tr}}_{{B}_{1}{B}_{2}}$ and $\:{{E}_{tr}}_{{B}_{3}{B}_{4}}$ indicates the energy transfer between a pair of adjacent cells.

The energy transfer between a pair of adjacent cells is calculated as:

16 $$\:{{E}_{tr}}_{{B}_{1}{B}_{2}}=\:\nabla\:{SOC}_{{B}_{1}{B}_{2}}\text{*}\:{V}_{c}\text{*}\:{C}_{c}$$

17 $$\:{{E}_{tr}}_{{B}_{3}{B}_{4}}=\:\nabla\:{SOC}_{{B}_{3}{B}_{4}}\text{*}\:{V}_{c}\text{*}\:{C}_{c}$$

The total power loss is calculated as follows:

18 $$\:{E}_{tpl}={E}_{trl}+{E}_{tsl}$$

Where, $\:{E}_{trl}$ and $\:{E}_{tsl}$ indicates the total resistive and total switching losses.

The total resistive loss is calculated as follows.

19 $$\:{E}_{trl}={P}_{{rl}_{PP}}\text{*}{t}_{b\:}$$

Where P_rlpp_ indicates total resistive loss per pair of cells and tb indicates the total balancing time of the battery pack.

Resistive losses per pair of cells are calculated as follows:

20 $$\:{P}_{{rl}_{pp}}={{I}_{b}}^{2}\text{*}R$$

Where I_b_ indicates balancing current, and R indicates resistance.

The balancing current is calculated as follows:

21 $$\:{I}_{b}=\frac{{V}_{cell}\text{*}D}{R}$$

The total switching losses are calculated as follows:

22 $$\:{E}_{tsl}={P}_{sl}\text{*}{t}_{b}$$

Where P_sl_ indicates power loss due to the switching action of switches and t_b_ represents balancing time.

The power loss due to the switching action is calculated as follows:

23 $$\:{P}_{sl}={V}_{c}\text{*}{I}_{b}\text{*}{t}_{s}\text{*}{f}_{s}$$

Where t_sw_ and f_sw_ indicate switching time and frequency. Energy Loss and Efficiency of Proposed Topologies are reported in Table 2.

## Simulation results and discussion

### Two-layer active equalisation topology

The proposed cell balancing model, shown in Fig. 4, is implemented and simulated using MATLAB/Simulink software. This model comprises three inductors (L_1_, L_2_, and L_3_, each rated at 10 mH) and six switches (S_1_-S_6_). Four batteries with a nominal voltage of 12.8 V, a cutoff voltage of 10.0 V, a fully charged voltage of 14.4 V, and a maximum capacity of 40 AH (36.2 AH at nominal voltage) form the battery pack. The initial states of charge (SOCs) for the batteries are set as B_1_ = 45%, B_2_ = 30%, B_3_ = 75%, and B_4_ = 60%, with an assumed SOC variation of 15% between cells for simulation purposes.

The control unit is responsible for generating the switching pulses required to activate the MOSFET switches based on the SOC readings of each cell. When a switch receives a pulse, it allows the corresponding inductor to charge (during the pulse-high phase) or discharge (during the pulse-low phase). This cycle continues at a duty cycle of 50% and a switching frequency of 10 kHz.

The balancing control algorithm, embedded within the control unit, generates switching pulses to equalize the SOC across the battery pack. These balancing initiates whenever the SOC difference between any two adjacent cells exceeds 0.5%. Based on the SOC readings, the control unit dictates the charging and discharging cycles of the inductors, thereby balancing the system.

**Fig. 15:**
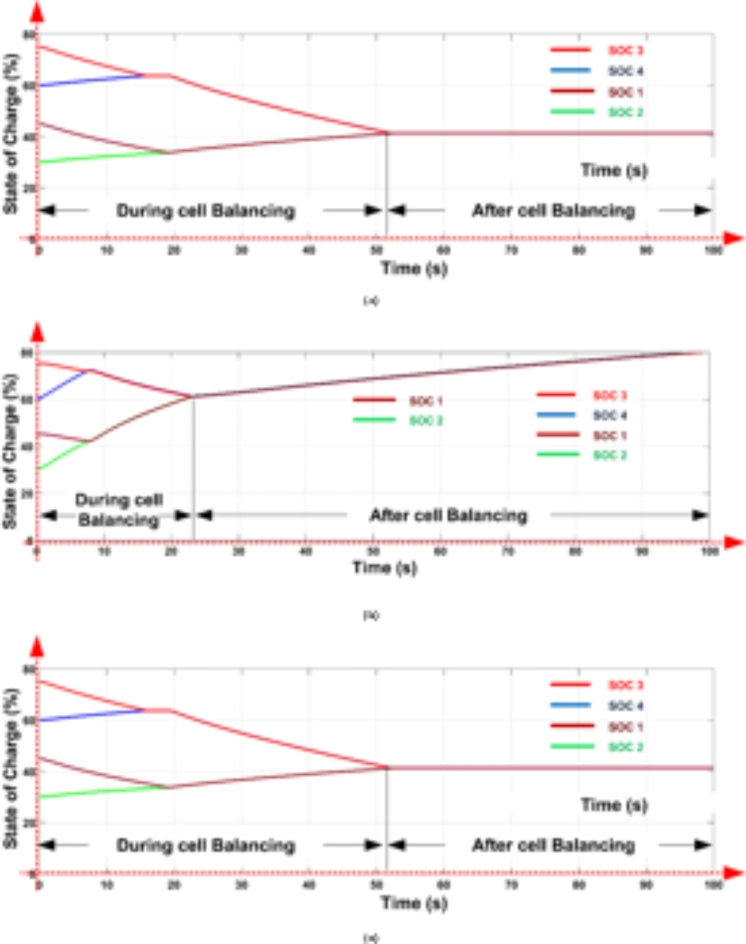
Cell Balancing During (a) Static Condition (b) Charging Condition and (c) Discharging Condition.

At the start of the balancing process, the control unit continuously monitors the SOC of each cell. Whenever the SOC difference surpasses 0.5%, it sends pulses to activate the respective switches. For example, the control unit activates switches S_1_ and S_3_, which charge inductors L_1_ and L_2_, transferring energy from B_1_ to B_2_ and from B_3_ to B_4_, respectively. When the SOCs of B_1_ and B_2_ stabilize at 34.5% and those of B_3_ and B_4_ at 64.5%, the control unit then activates switch S6 to transfer energy from the B_3_-B_4_ group to the B_1_-B_2_ group through L_3_, balancing the entire battery pack. This process, as shown in Fig. 15(a), is completed within 55 s, achieving an overall SOC of 43.5%.

The model was further tested undercharging and loaded conditions, as displayed in Fig. 15(b) and 15(c). During charging, the cells reached equilibrium within 25 s at a SOC of 62.27%. In the loaded condition, equilibrium was achieved within 55 s at a SOC of 43.5%, and the cells then began to discharge evenly to supply the load, as shown in Fig. 15(c).

### Optimized single-layer active equalisation topology

The proposed cell balancing model is implemented and simulated using MATLAB/Simulink software. This model includes three inductors (L_1_, L_2_, and L_3_, each with a rating of 10 mH) and four switches (S_1_-S_4_). Four Li-ion batteries are incorporated into the battery pack design, each with a nominal voltage of 12.8 V, a cutoff voltage of 9.6 V, and a fully charged voltage of 14.4 V. The maximum battery capacity is specified as 40 AH, with 36.2 AH available at nominal voltage. The initial states of charge (SOCs) for the batteries are set at B_1_ = 45%, B_2_ = 30%, B_3_ = 75%, and B_4_ = 60%, with an assumed SOC variation of 15% between cells for the simulation.

In this model, the control unit is programmed to generate switching pulses to activate the MOSFETs, which control the charging and discharging of the inductors. When a pulse is high, the MOSFETs allow the inductors to charge; when the pulse is low, the inductors discharge. A duty cycle of 50% and a switching frequency of 10 kHz are used to generate the pulses.

The balancing control algorithm within the control unit monitors the SOC of each cell and initiates balancing whenever the SOC difference between any two adjacent cells exceeds 0.5%. Based on SOC levels, the control unit directs the charging and discharging cycles of the inductors to achieve balance.

Initially, the control unit continuously reads the SOC of each cell to run the algorithm. When the SOC difference between two adjacent cells surpasses 0.5%, it sends pulses to the necessary switches to equalize the SOCs. For instance, switches S1 and S3 activate to charge inductors L_1_ and L_3_, transferring energy from B_1_ to B_2_ and from B_3_ to B_4_, respectively.

**Fig. 16:**
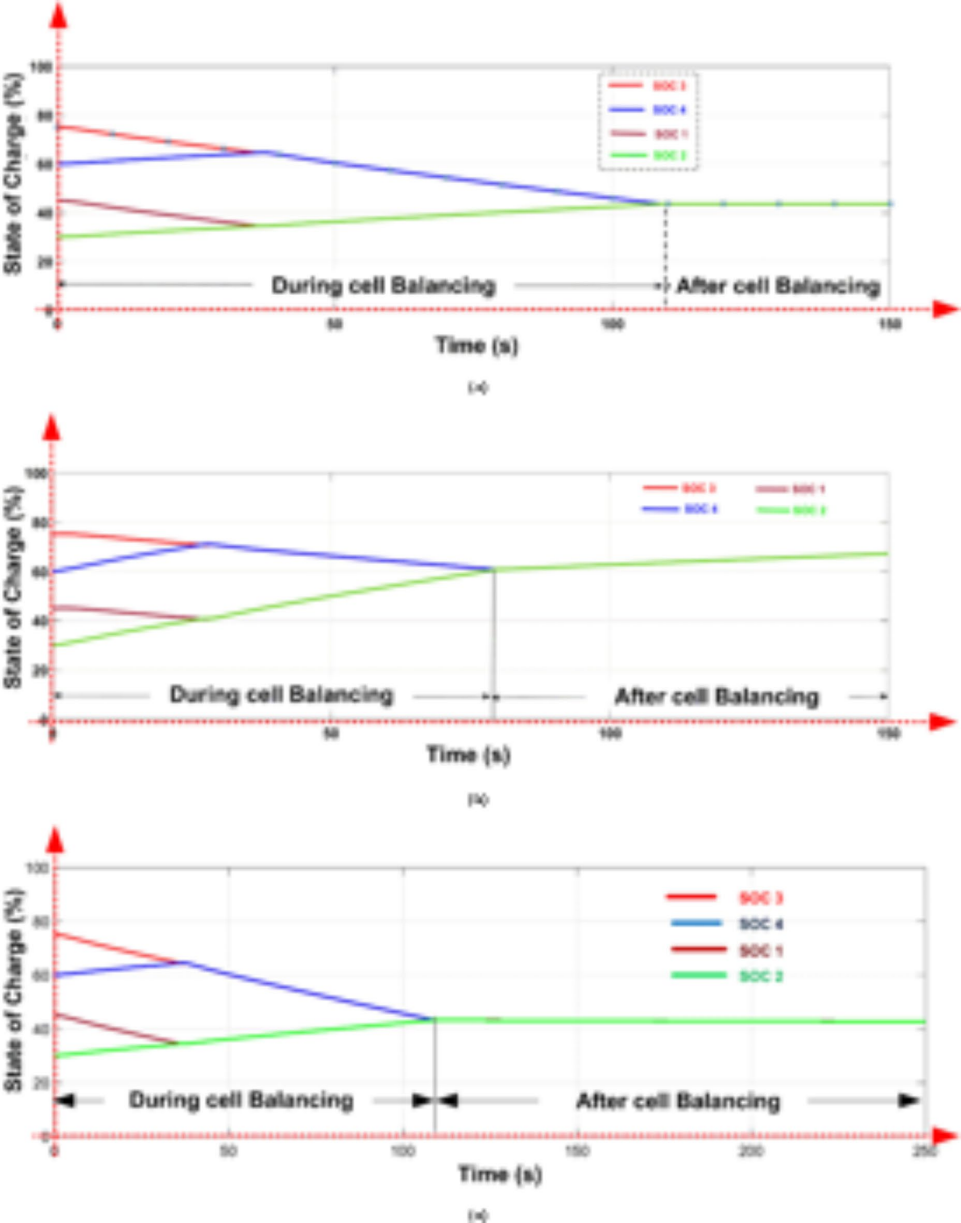
Cell balancing during (a) Static condition (b) Charging condition and (c) Discharging condition.

When cells B_1_ and B_2_ stabilize at a SOC of 34.5%, and B_3_ and B_4_ stabilize at 64.5%, as shown in Fig. 16, the control unit activates switch S_4_. This enables energy transfer from the B_3_-B_4_ cell pair to the B_1_-B_2_ pair through inductor L_2_, further balancing the battery pack. Figure 16(a) shows that the pack reaches full balance within 108 s at a SOC of 43.5%.

The model is also tested undercharging and loaded conditions, as shown in Fig. 16(b) and 16(c). During charging, equilibrium is reached at 80 s with a SOC of 60.05%. In the loaded condition, the cells balance within 108 s at a SOC of 43.5% and then discharge equally to supply power to the load, as shown in Fig. 16(c).

With this control unit-driven balancing strategy, the model maintains effective and safe SOC levels across the battery pack.

**Fig. 17:**
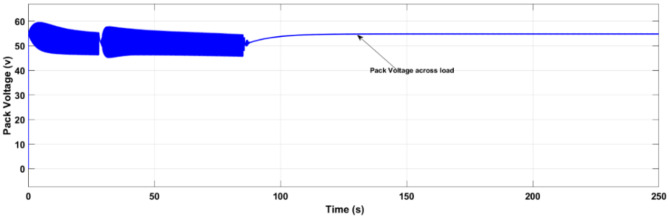
Simulation result of Pack Voltage.

**Fig. 18:**
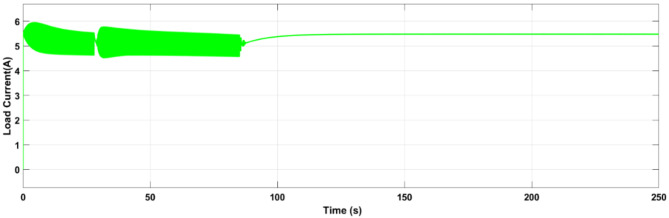
Simulation result of Pack Current.

All the switches are turned into an Off state by the controller when the battery pack is balanced. The load voltage and load current during the cell balancing of the battery pack are shown in Figs. 17 and 18.

**Table 2:** Energy loss and effiicency of proposed topologies.

Topologies	$\:{E}_{tsl}$ (Wh)	$\:{E}_{trl}$ (Wh)	$\:{E}_{tpl}$ (Wh)	$\:{E}_{tr}$ (Wh)	_c_ (%)
1 L MI ACB	0.000164	0.032832	0.032996	473.6	99.993
2 L ACB	0.000393	0.07866	0.079053	298.96	99.974

### Hardware results and discussion

**Fig. 19:**
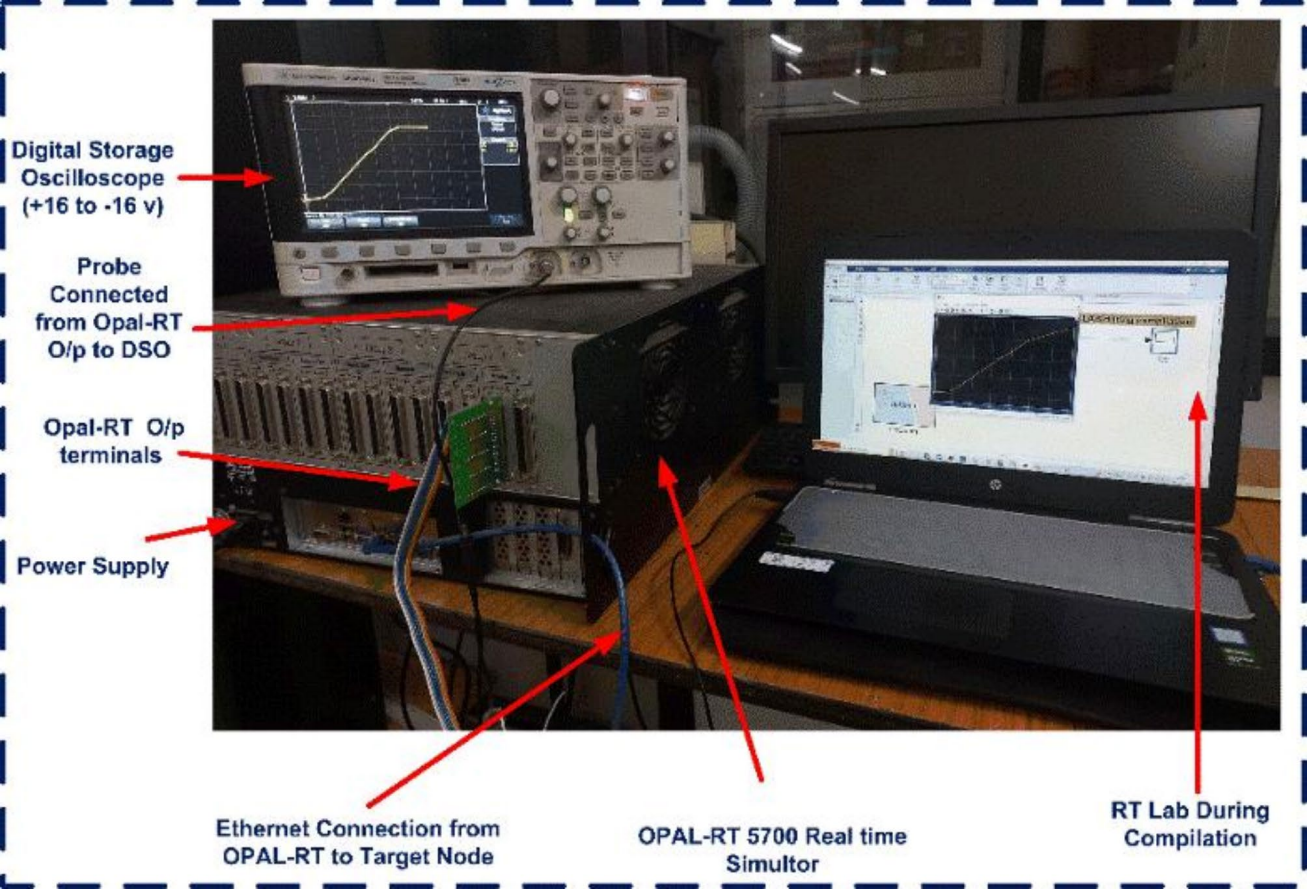
Experimental Setup using Opal-RT real-time simulator.

The proposed cell balancing topologies were developed using the Simulink tool in the MATLAB environment and tested on an OPAL-RT 5700 real-time Hardware-in-the-Loop (HIL) simulator. An Agilent Technologies DSO-X 2002 A oscilloscope was used to record the output results. The complete experimental setup is illustrated in Fig. 19, and Table 3 lists the components used for both the simulation and real-time HIL testing. Table 4 shows the specifications of the Opel-RT simulator.

**Table 3:** List of components.

Sl.no	Components	Quantity	Ratings
1	Li-Ion cells	4	12.8v, 40 AH
2	Inductors	3	10mH, 10ohms
3	MOSFET	6	
4	Load Resistor	1	10ohms

**Table 4:** Specifications of opel-rt simulator.

Device description	Specifications
Processor	Multi-core Intel Xeon
Memory	128 GB of DDR4 RAM
I/O Interfaces	CAN, Ethernet, and RS232/RS485
Operating System	RT-Linux or QNX
Software Tools	MATLAB/Simulink-2022b, RT-LAB

**Fig. 20:**
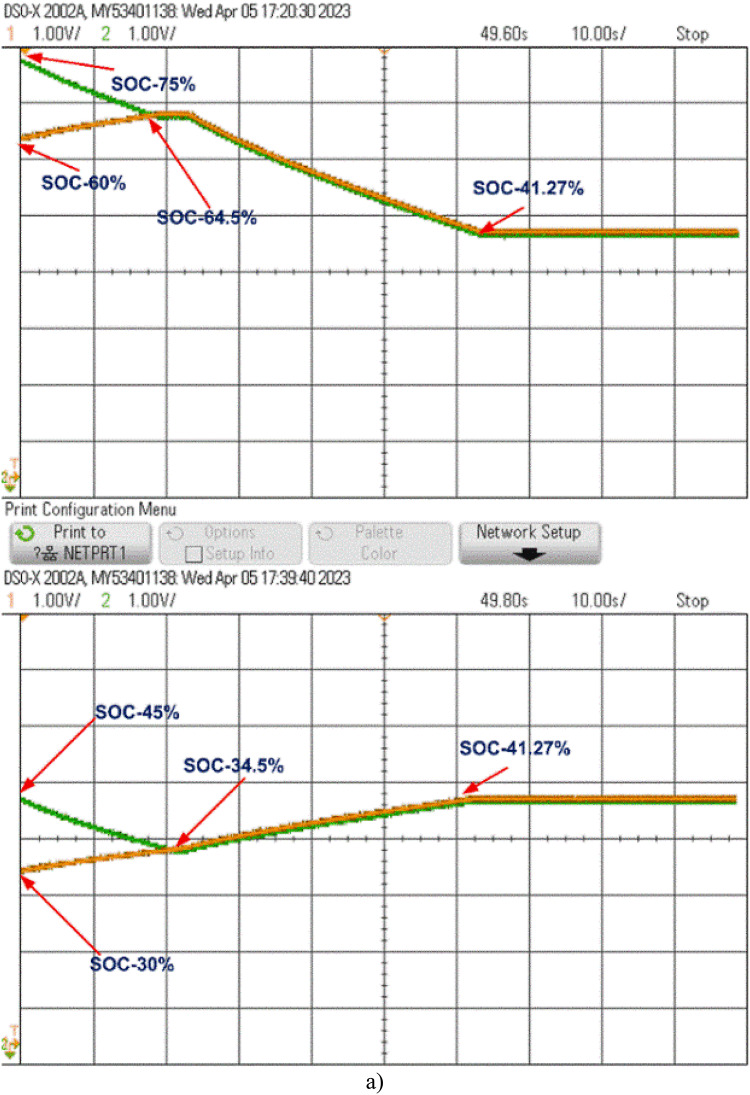

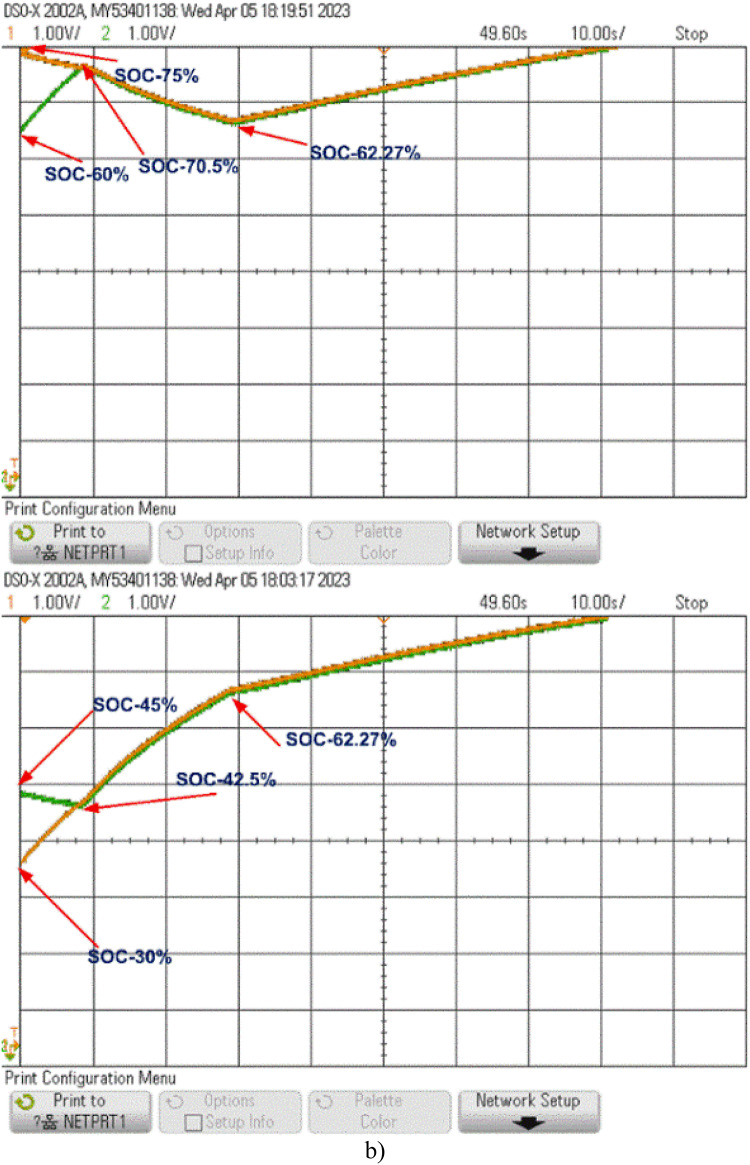

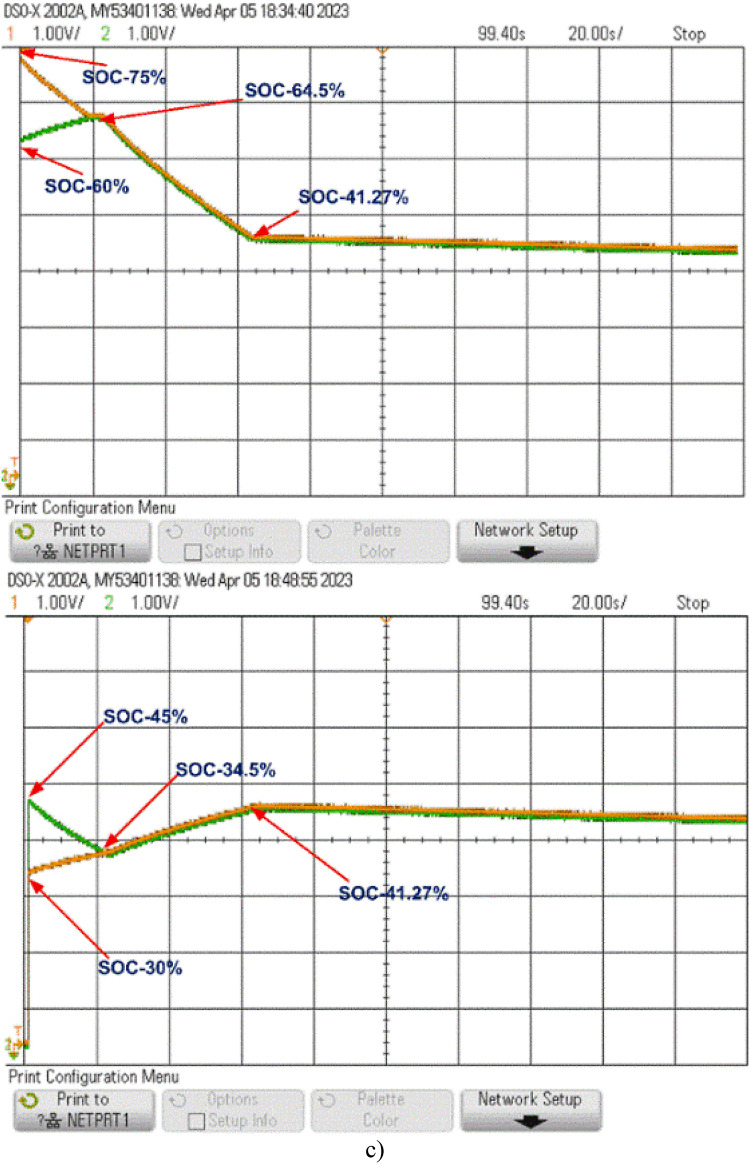
Cell balancing Experimental results using Opal-RT real-time simulator Two-layer inductor active equalisation topology during (a) Static condition (b) Charging condition and (c) Discharging condition.

**Fig. 21:**
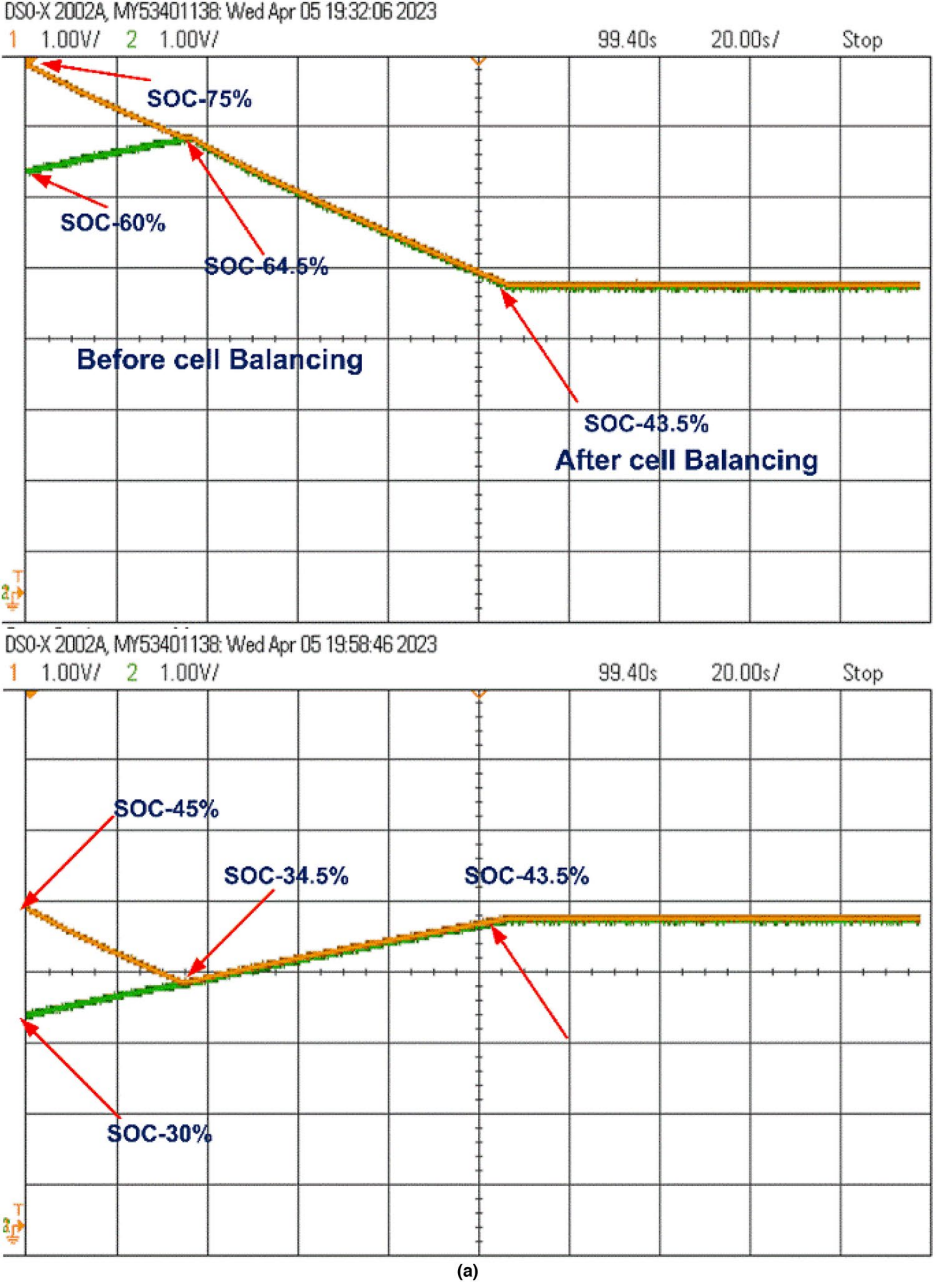

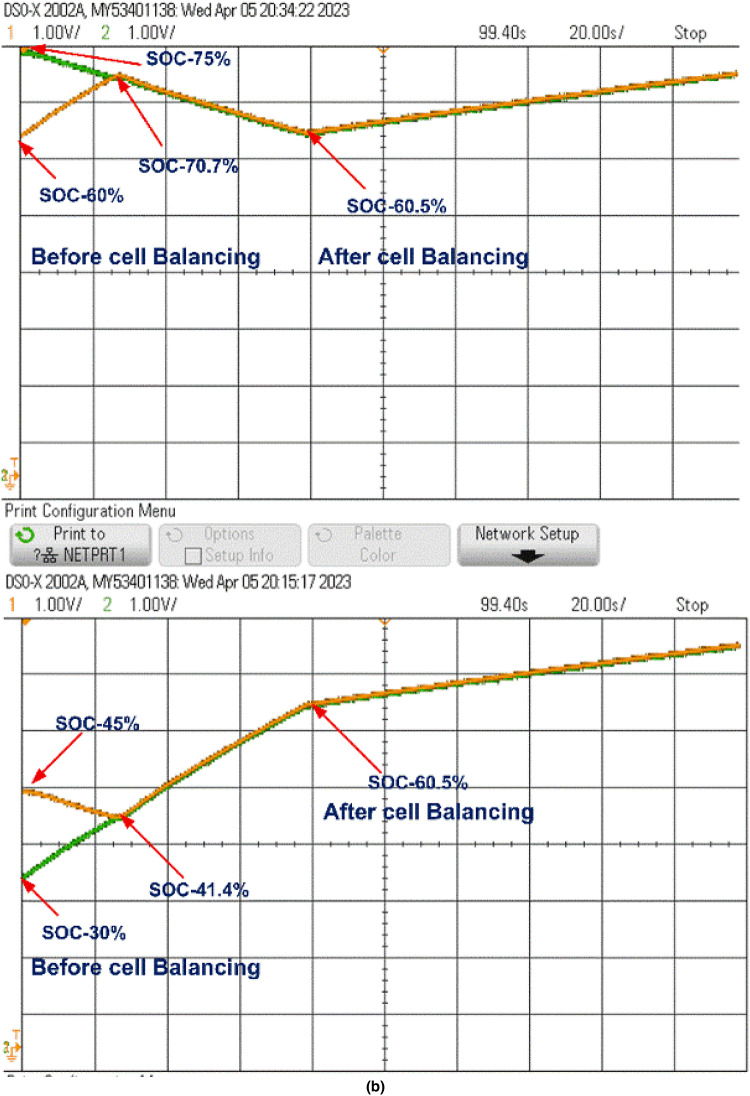

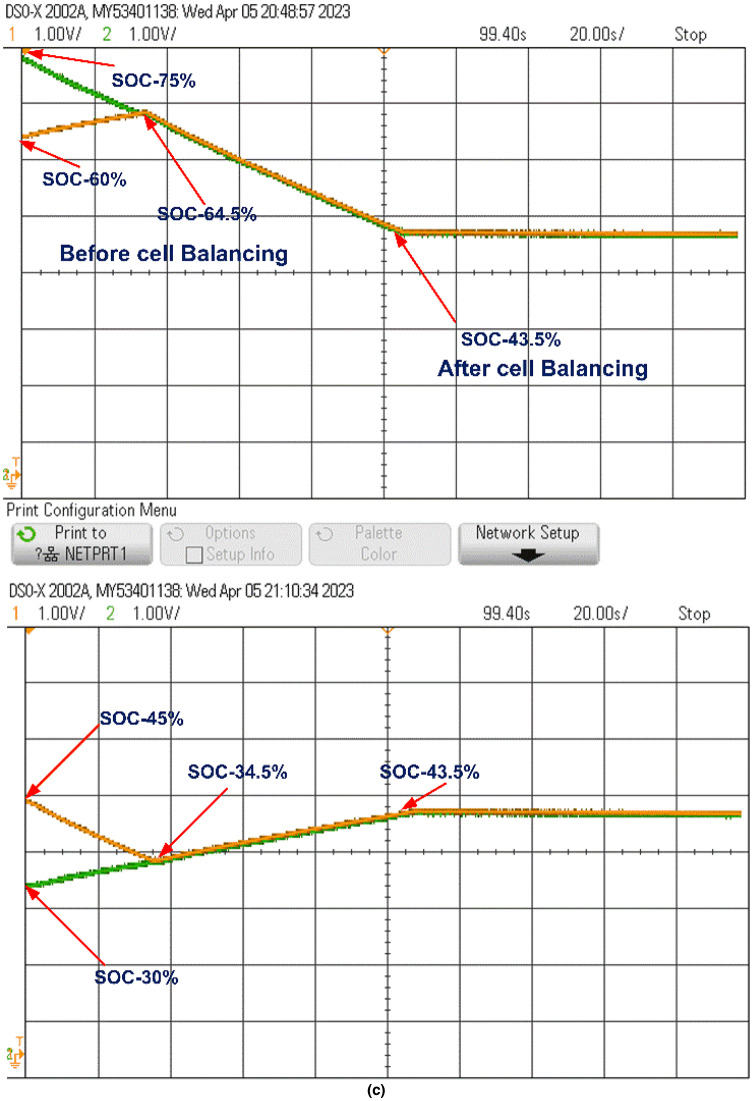
Cell balancing Experimental results using Opal-RT real-time simulator Optimized Single-layer inductor active equalisation topology during (a) Static condition (b) Charging condition and (c) Discharging condition.

**Fig. 22:**
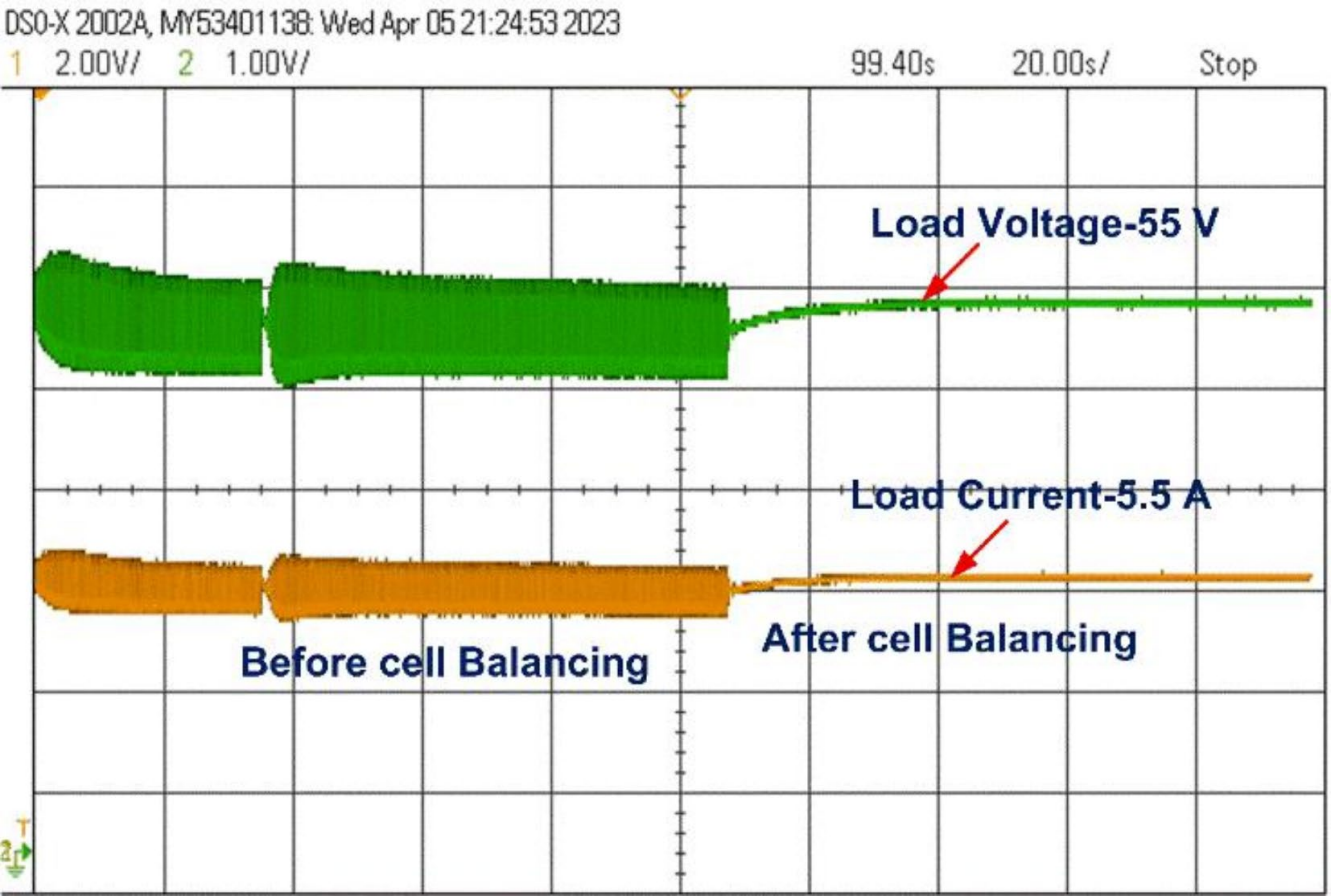
Load voltage and Load current during Cell balancing.

The cell balancing models shown in Figs. 4 and 7 were tested using the OPAL-RT HIL simulator with initial SOC values of B_1_ = 45%, B_2_ = 30%, B_3_ = 75%, and B_4_ = 60%. The real-time simulator performs both Model-in-Loop (MIL) and Software-in-Loop (SIL) testing, comparing results to expected values. If any discrepancies beyond threshold limits are detected, the system flags an error; otherwise, the test cases are deemed successful. During the SIL testing phase, the control unit in the simulation model is converted to a code-generated block, which replaces the original control unit with C-Code. This code is executed within the OPAL-RT’s real-time processor, allowing precise HIL testing. Once testing is completed, the oscilloscope collects the results, connected through a digital-to-analogue converter from the real-time simulator. The proposed model undergoes testing under static, charging, and discharging conditions, with the corresponding hardware results displayed in Figs. 20 and 21. Figure 22 shows the load voltage and current measurements during the cell balancing process.

In a two-layer inductor active equalization topology, all cells achieve balance within 85 s, reaching a SOC of 41.27%. After balancing, energy is distributed equally to the load. During charging, this topology demonstrates rapid balancing, achieving equilibrium in just 30 s a notable improvement over static and discharging conditions.

In the optimized single-layer setup, all cells reach balance at 110 s, stabilizing at a SOC of 43.5%. Post-balancing, energy is uniformly supplied to the load. During charging, the cells achieve balance within 80 s, faster than under static or discharging conditions.

In the Optimized single-layer inductor active equalisation topology, all the cells are balanced at 110 s with 43.5 present SOC and deliver energy to load equally. During charging all cells are balanced quickly at 80 s compared to the static and discharging condition. The model is connected to a resistive load (10 ohms), the load voltage and current are shown in Fig. 22.

### Comparative analysis

The proposed topologies are faster in balancing the battery pack compared to the existing research. In^39^an inductor-based cell balancing model with 4 cells, and 6 switches is proposed. The cell balancing process is designed from layer to layer in the model, it has taken 900 s to balance all the cells in the battery pack. In^40^a simulation model was designed to balance the four-cell battery pack with six switches using SimScape tools and state-flow. The results show that the time taken to balance the battery pack is 1046 s. In^41^a closed loop capacitor switching topology is used to balance the battery pack and nearly 1100 s is taken by the controller to balance the four-cell battery pack. The Dual Tired Capacitive Shuttling Method (DTCS) in^42^uses another layer of capacitors to speed up voltage equivalence. Energy can be transferred from the very first unit to the final unit in a battery pack of series-connected cells using this design. However, it puts a lot of strain on the capacitors, and equilibrating the voltages of the cells takes a long time (4000 s) and will take longer if more cells are added. The parallel structured switching capacitor equalizer (PSSC) topology in^43^ allows energy to be transferred from any of the cells in the battery pack to any other cell, but it requires a longer time (4500 s) to achieve voltage equality. The proposed approach to balancing topologies is superior to DTCS and PSSC topologies. The comparative analysis is shown in Table 5. Table 6 shows the comparison between Simulation and Opal-rt results.

**Table 5:** Comparative analysis of proposed topologies with existing methods.

Sl. No.	Existing technology	No. of Cells	No. of Switches	No. of inductors /Capacitors	Balancing time(sec)
1	[75]	4	8	6	1100
2	[76]	4	8	4	4000
3	[77]	4	8	4	4500
4	[78]	4	6	3	1046
5	[79]	4	6	3	900
6	[82]	10	6	8	6200
7	[83]	8	8	6	4500
8	[84]	4	8	6	6200
9	[85]	12	8	10	4200
10	Two-layer Equalisation topology	4	6	3	54
11	Optimized Single- layer Equalisation topology	4	4	3	108

**Table 6:** Comparative analysis between simulation and opal-rt simulator results.

Condition	Algorithm	Simulation result/ Balancing time(sec)	Real-time HIL result/ Balancing time(sec)
Static state	2 L MI-ACB	54	65
	Single layer MI-ACB	108	110
Charging State	2 L MI-ACB	22	18
	Single layer MI-ACB	80	80
Discharging state	2 L MI-ACB	54	65
	Single layer MI-ACB	108	110

## Conclusion

A MATLAB/Simulink model was developed in this work to assess the efficiency and effectiveness of active balancing in an eight-cell LIB battery pack. The model focused on two-layer (2 L) and single layer Multi-Inductor Active Cell Balancing (MI-ACB) circuits. The simulation results demonstrated notable improvements in SOC balancing, with reduced balancing times. Specifically, the 2 L MI-ACB successfully balanced all cells within 54 s in simulation and 65 s in real-time hardware-in-the-loop (HIL) testing under static conditions, resulting in an overall SOC of 43.5%. In comparison, the single layer MI-ACB required 108 s in simulation and 110 s in HIL testing to achieve a similar balance. The Single layer MI-ACB demonstrated an efficiency of roughly 99.993%, whereas the 2 L MI-ACB exhibited an efficiency of 99.974%. The 2 L MI-ACB architecture exhibited enhanced speed and efficiency in balancing the battery pack in comparison to current designs, but the single layer MI-ACB offered a cost-efficient option with a reduced number of switching devices. Potential future research could focus on optimizing parameter values, such as minimizing inductance and resistance and increasing the switching frequency to enhance efficiency and reduce the time required for balancing. The results highlight the capability of sophisticated MI-ACB topologies to improve the efficiency of battery management systems, laying the groundwork for continuous advancements in battery technology.

## Data Availability

The data used to support the findings of this study are included in the article.
